# Freshness and Spoilage Patterns of Wild and Farmed Tropical Fish Species with Major Commercial Importance Originating from Saudi Arabian Waters

**DOI:** 10.3390/foods14040690

**Published:** 2025-02-17

**Authors:** Kriton Grigorakis, Dimitra Kogiannou, Mado Kotsiri, Ioannis Kleidas, Paulo H. de Mello, Salaheldeen Habiballah, Ali Alshaikhi, Youssef S. Alhafedh, Asaad H. W. Mohamed

**Affiliations:** 1 Hellenic Center for Marine Research (HCMR), Institute of Marine Biology, Biotechnology & Aquaculture, 19013 Anavyssos, Greece; dkogiannou@hcmr.gr (D.K.); mkotsiri@hcmr.gr (M.K.); i.kleidas@hcmr.gr (I.K.); 2 Kaust Beacon Development, King Abdullah University of Science and Technology, Thuwal, Jeddah 23955, Saudi Arabia; paulo.demello@kaust.edu.sa (P.H.d.M.); salaheldeen.habiballah@kaust.edu.sa (S.H.); asaad.mohamed@kaust.edu.sa (A.H.W.M.); 3 Ministry of Environment, Water and Agriculture, King AbdulAziz Rd., Riyadh 11195, Saudi Arabia; ali.alshaikhi@mewa.gov.sa (A.A.); yhafedh@nfdp.gov.sa (Y.S.A.)

**Keywords:** QIM, K-value, fish quality, microbiological changes, seafood, sensory, wild, farmed, chilling

## Abstract

Ice-stored farmed barramundi (*Lates calcarifer*), snubnose pompano (*Trachinotus blochii*) and sobaity bream (*Sparidentex hasta*), as well as wild-caught cobia (*Rachycentron canadum*), coral trout (*Plectropomus leopardus*), giant trevally (*Caranx ignobilis*), milkfish (*Chanos chanos*) and mangrove red snapper (*Lutjanus argentimaculatus*), were compared for their freshness/spoilage using sensory, chemical and microbiological methods. Quality Index Method schemes were developed to determine alterations in the sensory freshness. The shelf lives ranged from 8 (coral trout) to 18 days (sobaity bream). The farmed species always exhibited a significantly longer shelf life than the wild-caught species. The adenosine triphosphate (ATP) breakdown followed different patterns in the studied species. The K-values at the time of sensory rejection ranged from 30 to 80% depending on the species, while the microbial load reached or exceeded a level of 6 log cfu/g. Although the shelf life duration was dependent on the origin of the fish (wild or farmed), the ATP breakdown scheme, as well as the K-values and microbial loads at the time of rejection, were species-dependent and independent of the origin.

## 1. Introduction

Freshness is considered one of the four pillars of fish quality, along with safety, traceability and authentication [1]. Fish is a food item that is characterized by its high perishability; this, in practice, is translated to a rejection rate of 10–50% of all products due to post-slaughter spoilage. Therefore, freshness and spoilage are of capital importance in the commercial chain of fish. Three main postmortem processes appear to happen, namely enzymatic autolysis, oxidation and microbial degradation, and these are responsible for the transformation of certain substances in fish tissues that result in spoilage. In fresh ice-stored fish, autolysis and microbial degradation prevail, while oxidation is more pronounced in frozen storage [2,3].

Different species exhibit different spoilage patterns [2,4] and speeds; therefore, individual monitoring of freshness reduction/spoilage is required to understand these patterns. The study of freshness and its reduction requires tools such as sensory, chemical and microbiological evaluations [2,4,5]. Among the various methods for sensory freshness evaluation, the Quality Index Method (QIM) seems to be the preferred tool, as it is species-specific and highly analytic, thus providing a numerical score that is more representative of the spoilage stage [5,6]. While significant attention has been paid to the freshness and spoilage of fish species that are popular in the European, Japanese and US markets, scarce data are available for popular tropical species that are highly important in other Asian and African fish markets.

Barramundi (*Lates calcarifer*) is a species mainly originating from the Indian and Pacific Oceans; it is also widely farmed, with a global production of 154,280.7 (FishstatJ, 2024 v4.04.00, https://www.fao.org/fishery/en/statistics/software/fishstatj, accessed on 20 December 2024) metric tons in 2022 and market sizes ranging from 500 g to 2 kg. Cobia (*Rachycentron canadum*) is a globally distributed warm-water species, with a global harvesting volume of 19,147.73 (2022) and global production of 35,613.45 metric tons in 2022 (FishstatJ, 2024). China and Taiwan are the main producers, with market sizes of 5–10 kg and net exports of its fresh form reaching USD 30 million in 2020 [7]. Coral trout species (*Plectropomus* sp.) are sold in the local Saudi fish market, with a global production of about 250 metric tons; however, their value is ranked highest among the fish that are traded in the local Red Sea markets, exceeding 25 USD/kg [8]. The giant trevally (*Caranx ignobilis*) is an important species with Indo-Pacific distribution, a global harvesting volume of 109,000 metric tons in 2017, and farming attempts taking place in recent years in the Philippines (https://www.fao.org/fishery/aquagris/en/aqgr/phl__nxi?status=pending, accessed on 20 December 2024). The milkfish (*Chanos chanos*), an herbivorous species that is commercialized in market sizes of 250–500 g, accounted for a global aquaculture production exceeding 1,196,017.29 metric tons in 2022 (FishstatJ, 2024). It is highly valued in Asian markets and has a long and robust farming tradition that started in Indonesia, the Taiwan Province of China and the Philippines four to six centuries ago, but a significant number of wild fishery catches also occur [7]. The mangrove red snapper (*Lutjanus argentimaculatus*) is a widespread Indo-Pacific species with significant market importance in the region but is never found in significant quantities, with annual production reaching approx. 15,000 tons from fisheries and 5308 (2022) from aquaculture, according to the species factsheet of the FAO (https://www.fao.org/fishery/en/aqspecies/3134/en, accessed on 20 December 2024; FishstatJ, 2024). The snubnose pompano (*Trachinotus blochii*) is a widely farmed species in China, India, Indonesia, the Philippines, Taiwan, Thailand and Vietnam, with significant importance in these markets. There is high demand for this species, including in the US market, with a dockside price of USD 8 in international markets, as reported in the respective FAO factsheets (https://www.fao.org/fishery/en/culturedspecies/trachinotus_spp/en, accessed on 20 December 2024). The sobaity bream (*Sparidentex hasta*) is a species with significant importance in fish farming in the Arabian Peninsula, a marketable size of 400 g and very high market potential. The latest estimation of its aquaculture production was 241 metric tons in 2022 (FishstatJ, 2024).

Despite the capital economic importance of these species, which is outlined herein, there is a significant lack of knowledge regarding their freshness and spoilage patterns. Such knowledge is important in ensuring their quality during commercialization.

Within this context, the aim of this study was to systematically assess the freshness and spoilage of the aforementioned commercially important fish originating from national aquaculture and fisheries in the Saudi Arabian market and, subsequently, to compare their freshness/deterioration. For this purpose, the chemical, microbial and sensory spoilage of eight different species were analytically studied, and the individual characteristics of their spoilage patterns were assessed.

## 2. Materials and Methods

### 2.1. Fish

In total, eight locally (Saudi Arabia) marketed fish species were studied, selected on the basis of their market importance, availability and lack of data on their freshness and spoilage patterns. The characteristics of the studied fish, including their average weights, appear in Table 1.

All the analyzed fish were whole, unprocessed or fresh and provided from the Central Fish Market in Jeddah. They were freshly landed or slaughtered (farms) and remained in Styrofoam boxes with ice thereafter. The farmed fish were slaughtered, packed with ice and transferred to the market within a day, thus ensuring the maintenance of absolute freshness until market distribution. The wild-caught fish were iced on the vessel, although no accurate estimation of the slaughter time was provided. For each species, one batch of 15 fish was selected in order to have adequate samples for studying the sensory freshness and developing the QIM schemes; subsequently, a second batch with an equal number of individuals of each species was used for scoring in the developed QIM, analyzing their chemical freshness and measuring the microbial spoilage.

The fish were transferred to King Abdullah University of Science and Technology (KAUST) facilities for sampling and a sensory freshness evaluation during the first 8 days of storage. To assess the late spoilage stages, the sampled fish were subjected to identical analyses at the Hellenic Centre for Marine Research (HCMR) facilities in Greece. Storage always occurred under refrigeration in cold rooms (4 °C). The permanent presence of ice ensured a temperature of 0 °C around the fish while at the same time reducing the bacterial load and retaining moisture in the fish skin. Therefore, periodic ice replacement and continuous ice presence were ensured throughout the experimentation.

For all species, the sensory freshness, microbial status and chemical freshness were evaluated at specific time intervals from the time 0 (the time of obtainment) to a time when the fish had reached/passed the acceptability limit (the shelf life). The acceptability limit was detected by means of human senses, i.e., the perceived sensory freshness.

### 2.2. QIM

The QIM is a sensory freshness evaluation tool that differs for each species. Depending on the individual sensory changes that a species undergoes during freshness reduction/spoilage, a system of demerit scores is developed based on the changes in specific features, as perceived organoleptically.

With the exception of cobia, where QIM schemes are already available [9], the QIM schemes for the rest of the species had to be developed from scratch. For the development of a QIM, changes in the external appearance/visual quality, external smells and texture were analytically monitored. For each feature, these changes were evaluated from the start, namely the first day after slaughtering for farmed fish or the first day after purchase for wild ones, until the point when a fish passed its shelf life and could not be commercialized (i.e., beyond the sensory acceptability limit). A total of 3–5 specimens were used at each sampling point to evaluate freshness and determine their characteristics and the changes that they underwent.

For cobia, the already existing scheme was generally followed, with minor alterations after the preliminary screening procedures.

Three assessors were used, each of whom scored 3 individual fish (for each species) at every sampling point, based on the respective previously developed QIM schemes. The total scores that they received were averaged to establish the freshness score at each time interval. Standard deviations and the two-tail Pearson correlation with days of ice storage were used to check the validity of the created schemes.

### 2.3. Chemical Freshness: K-Value and ATP Breakdown Products

The adenosine triphosphate (ATP) breakdown products were analyzed using high-performance liquid chromatography (HPLC), and K-value determination was performed to evaluate the chemical freshness and determine the shelf life in chemical terms. The determination of ATP breakdown products, which include ATP, adenosine diphosphate (ADP), adenosine monophosphate (AMP), inosine monophosphate (IMP), inosine (Ino) and hypoxanthine (Hx), took place after extraction from dorsal white muscle tissue. A quantity of 5 g was homogenized on ice with 25 mL of perchloric acid (PCA; HClO_4_, 0.6 Μ) for 2 min. Subsequently, centrifugation at 4 °C and 7000× *g* for 5 min took place, and 10 mL supernatant was obtained. The pH was adjusted to 6.7–6.9, with KOH 1 Μ initially and 0.1 Μ subsequently for the fine adjustment.

The HPLC determination of the ATP breakdown products took place according to the previously published methodology in [10]. The method for the separation and detection of ATP metabolites was originally developed and validated by Ryder [11]. The apparatus that was used combined a Waters 600 Pump, a 600 Pump system Controller, a Waters 717 Plus Autosampler set at a 10 °C injection temperature, a Waters 2487 UV detector set at 254 nm and Empower Chromatography Software v5.00.00.00 (Waters, Milford, MA, USA). The mobile phase consisted of 0.4 Μ KH_2_PO_4_ and 0.06 Μ K_2_HPO_4_ in HPLC water. The flow was set at 1.5 mL/min. An aliquot of 5 μL was injected. The chromatographic column was a C18, using 5 μm, 100 RP and 4 × 250 mm (Phenomenex, Torrance, CA, USA). The total running time per sample was 20 min.

Subsequently, the identification of individual ATP metabolites took place, and we determined the K-value as follows:

$$K\;value\%=100\times \frac{Ino+Hx}{ATP+ADP+AMP+IMP+Ino+Hx}$$

### 2.4. Microbial Spoilage

Microbial spoilage was evaluated following previously published methodologies [12,13], with minor modifications. Briefly, 10 g from the dorsal muscle of each fish was placed into stomacher bags with 90 mL of sterile MRD (maximum recovery diluent: 8.5 g/L of NaCl and 1.0 g/L of bacteriological peptone) and homogenized for 1.5 min using a stomacher. Then, volumes of 0.1 mL from the serial dilutions in the MRD were spread onto the surface of water plates to count the agar in order to enumerate the total viable counts (TVCs) and incubated at 27 °C for 48 h. Samples of 1 mL of serial dilution in the MRD were used for the pour plate technique for the enumeration of (a) H_2_S -producing bacteria (presumably *Shewanella* sp.) on iron agar by counting only black colonies after incubation at 27 °C for 48 h, and (b) Enterobacteriaceae (*E. coli*) on Violet Red Bile Glucose agar, which was incubated at 37 °C for 24 h. The results were expressed as the mean log cfu/g ± standard deviation of 3 replicates (3 fish per species).

### 2.5. Statistical Analysis

For all fish species and for every measured factor, the average and standard deviations were calculated. Differences in the means in the K-values and the ATP breakdown products were statistically tested by performing an analysis of variance (ANOVA), followed by Tukey’s significant difference test (IBM SPSS Statistics Version 26 software). The levels of significance were set at *p* = 0.05 (5%).

## 3. Results and Discussion

The QIM is considered the most widely used and reliable method for sensory freshness evaluation, not only for ice-stored fish but also for a variety of both fresh and frozen seafood and other muscular foods [4,5,14,15,16,17]. To accomplish the aim of this study, new QIM schemes were designed for most of the tested fish species. For cobia, the already developed scheme [9] was slightly modified based on the observations of the present study. The analytic QIM schemes for each species can be found in Supplementary Tables S1–S8 (Supplementary-material QIM schemes). Representative photos of the fish in the initial (fresh) and final (at the acceptability limit) stages are provided in Supplementary Figures S1–S8.

**Figure 1:**
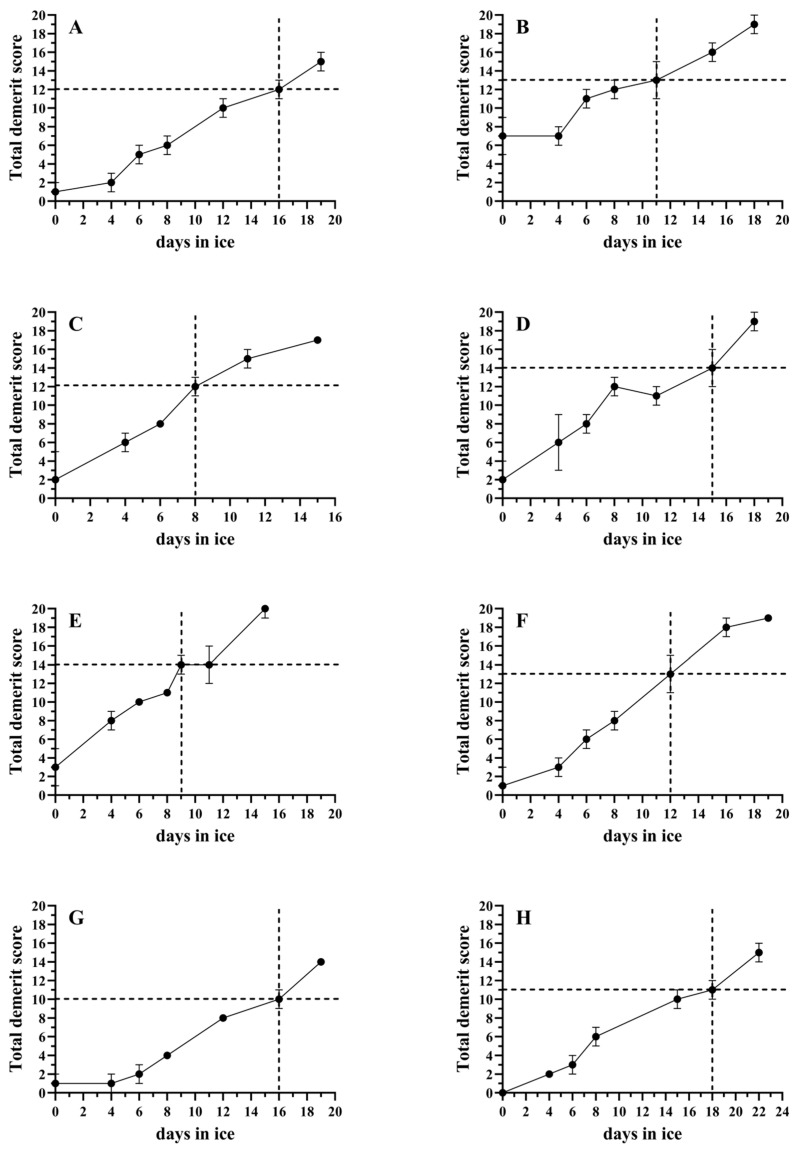
QIM sensory freshness evaluations of the studied species during ice storage. (**A**) Barramundi, (**B**) cobia, (**C**) coral trout, (**D**) giant trevally, (**E**) milkfish, (**F**) mangrove red snapper, (**G**) snubnose pompano and (**H**) sobaity bream. The vertical dotted line in the diagrams marks the acceptability limit (in days), while the dotted horizontal line indicates the QIM score that the fish received at this point. The bars indicate the standard deviations (n = 3).

Following the evaluations using the developed QIM schemes, each of the studied species was scored at various intervals during ice storage (Figure 1). For snubnose pompano, the total demerit score at the acceptability limit was 10; for sobaity bream, it was 11; for coral trout and barramundi, it was 12; for cobia and mangrove red snapper, it was 13; and for milkfish and giant trevally, it was 14.

In general, the spoilage schemes of the different fish species have commonalities, namely, the resolution of rigor mortis and softening of muscle tissue, the loss of skin color intensity, the development of undesirable odors in the gills and skin, and the loss of eye convexity and shine [2]. Interestingly, the present study revealed individualities in the tested species. The coral trout initially exhibited soft muscles, with muscle hardening occurring toward the later stages of spoilage. The milkfish exhibited a remarkable black discoloration in the belly area and a mass loss of scales. The mangrove red snapper exhibited excessive discoloration patches in the red skin as spoilage progressed. Freitas, Vaz-Pires and Câmara [15] indicated that the main disadvantage of QIM is its species specificity, which leads to the necessity of individually developing different schemes. The aforementioned study also highlighted the limitations relating to the variability of the specimens that are assessed and the assessors’ judgments and proposed a validation scheme for QIM. However, it was stated that these procedures add to the complexity of the QIM establishment process. The low standard deviations of all QIM scores (Figure 1) and the QIM correlations with days of ice storage were >0.98 in all cases for each species, indicating the proper function of the herein-developed schemes.

Based on the QIM sensory evaluation, the acceptability limit for each species was evaluated, and the awarded demerit scores appear in Figure 1. Among the studied species, coral trout and milkfish were the most perishable ones, with shelf lives of 8 and 9 days, respectively, while sobaity bream was found to be the most durable, with an 18-day shelf life. These were followed by the remaining farmed species (barramundi and snubnose pompano), which had a shelf life of 16 days of ice storage. The shelf life of the aforementioned farmed species was quite similar to that of gilthead seabream, which is in the range of 15–17 days [10,18,19], and red seabream.

The significantly longer shelf life of the farmed species compared with that of their wild counterparts can be explained by the use of controlled slaughtering conditions and an undisrupted cold chain. Additionally, the lower amount of stress that occurs during slaughtering compared with the stress that wild fish undergo during the fishing process may also be a factor. The longer shelf life of farmed fish compared with their wild counterparts of the same species has been previously reported and is in accordance with the present observations [20]. The shelf life of cobia that was determined herein was significantly shorter than that mentioned in [9] for the same species, which was 15 days of ice storage. A reasonable explanation for this is that the individuals that were studied herein were captured from the wild, unlike those in the aforementioned study, which were farmed. In a study referring to a grouper species of the same genus (*Plectropomus* sp.) as the coral trout, a shelf life of 18 days of ice storage was indicated when the fish were slaughtered and immediately stored in ice [21]. However, in the same study, a mere two-hour delay in icing, i.e., storage at an ambient tropical temperature, resulted in a shelf life of 8 days. This duration matches the herein-found shelf life for coral trout.

A basic autolytic procedure that occurs after fish death is the anaerobic metabolism of glycogen (due to the absence of respiratory function), which produces ATP. This, unlike in living fish, cannot be re-generated to provide energy to the cell. The ATP is inevitably further metabolized into other substances [22,23]. The initial pathway steps from ATP to IMP are very quick—almost immediate—and IMP is the first intermediate product that accumulates and is further gradually metabolized [24].

It has been shown that the ATP breakdown is dictated by degradation pathways, including endogenous enzymes, as well as microbial mechanisms of action. The enzymes that are produced by the spoilage microorganisms are the ones that are mainly responsible for the degradation of IMP and related products during the mid- to late stage of the storage of fish [23]. The two final products, i.e., Ino and Hx, gradually increase as fish spoilage proceeds. The measurement of these individual products’ concentrations, as well as their ratio, namely, the K-value, provides useful information on the freshness/spoilage stage of fish. It has been found the K-value is the most reliable among the chemical indices and that it correlates better with the sensory freshness of fish [19,25].

**Figure 2:**
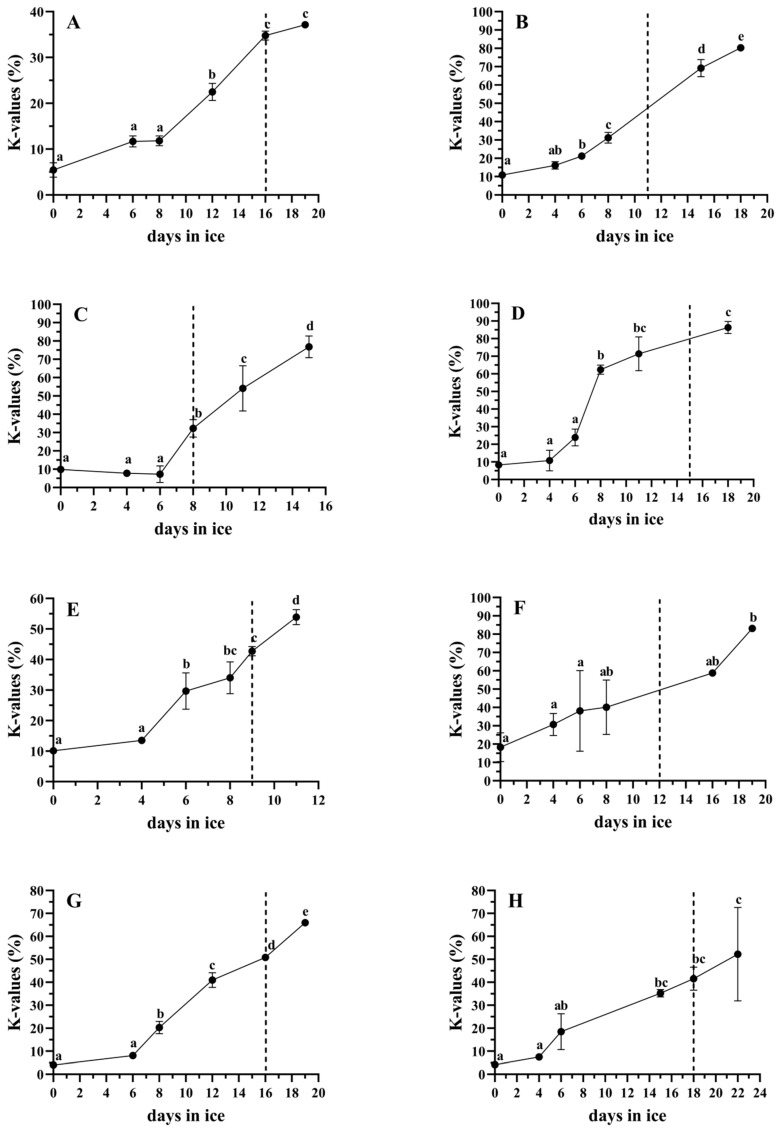
K value changes during ice storage of (**A**) barramundi, (**B**) cobia, (**C**) coral trout, (**D**) giant trevally, (**E**) milkfish, (**F**) mangrove red snapper, (**G**) snubnose pompano and (**H**) sobaity bream. The dotted line represents the day that the acceptability limit was reached (end of shelf life). Different letters represent statistically significant differences (*p* < 0.05). The bars represent the standard deviations (n = 3).

The K-values of the eight studied species are presented in Figure 2. The red dotted line marks the shelf life duration and the K-values of the species at the end of their shelf life. For most of the species, K-values of around 40–50% characterize fish that are at their acceptability limit. However, there are exceptions, such as in the giant trevally, which had a K-value of around 80% at the acceptability limit, or the barramundi and coral trout, which exhibited lower K-values of around 30% at the end of their shelf lives. Similarly to our results, a K-value of 40% has been previously mentioned to generally describe spoiled fish [26].

**Figure 3:**
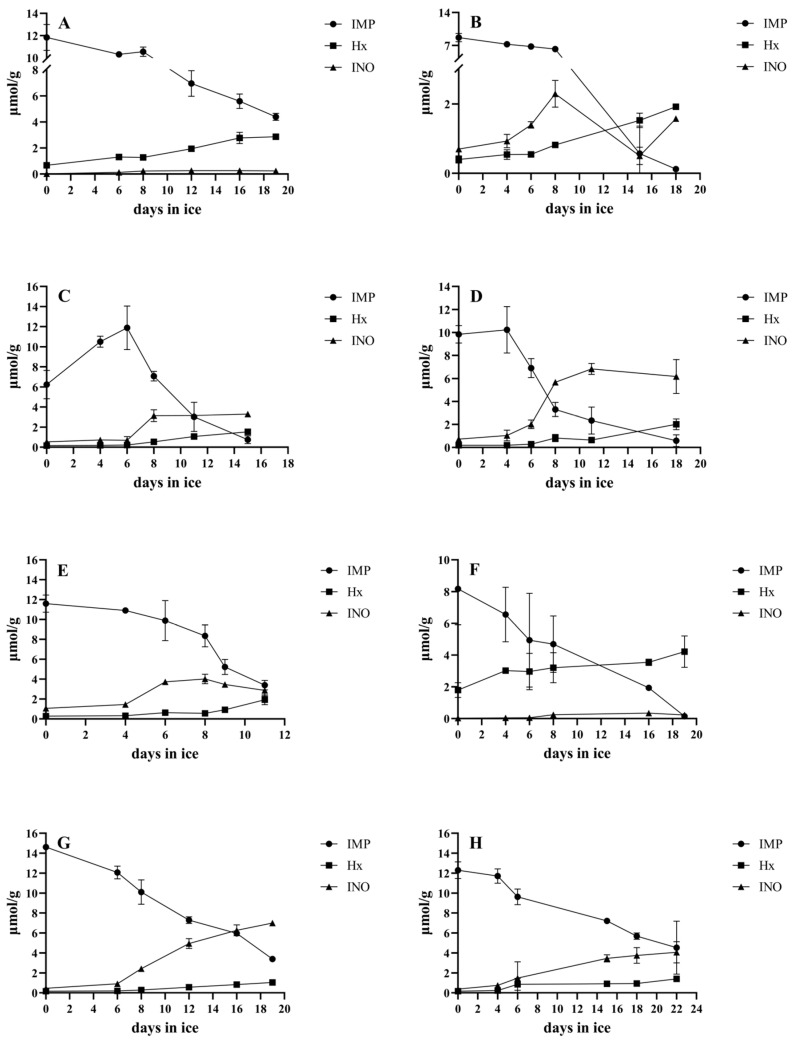
IMP, Ino and Hx concentrations of the studied species during ice storage. (**A**) Barramundi, (**B**) cobia, (**C**) coral trout, (**D**) giant trevally, (**E**) milkfish, (**F**) mangrove red snapper, (**G**) snubnose pompano and (**H**) sobaity bream. The bars represent the standard deviations (n = 3).

The changes in the concentrations of the three basic individual ATP metabolites, namely the IMP, Ino, and Hx, for the studied species are presented in Figure 3.

Although the speed of ATP breakdown and the concentrations of ATP metabolites seem to be affected by various factors, including the season, aquaculture pattern, storage temperature, stunning method, and harvest treatments like bleeding [23,27], the pattern of ATP metabolite changes is strongly species-specific [24]. In our study, we found that snubnose pompano and sobaity bream had some commonalities in their ATP breakdown patterns. These included slow gradual IMP breakdown and subsequent Ino formation, while the Hx levels remained low with a small and delayed increase. The final concentrations of Hx in their muscle never surpassed 1–1.5 μmoL/g. The Ino levels increased significantly (*p* < 0.05) after the 8th day of ice storage in snubnose pompano and on the 14th day in sobaity bream. These two species can be characterized as non-Hx forming species. A similar ATP breakdown pattern was described for gilthead seabream [19]. In contrast, the mangrove red snapper was found to be a characteristic of Hx-forming species, with Hx increasing at high levels from the beginning and further increasing with spoilage. Barramundi also seemed to be a Hx-forming species but with a more gradual Hx increase. These two species both have high levels of Hx but also very low and fluctuating levels of Ino.

In the giant trevally, Ino gradually increased, peaked and then plateaued around day 10 (with a significant change, *p* < 0.05), followed by a very slow decrease to the level of Hx thereafter. The Hx only increased significantly (*p* < 0.05) on day 18, i.e., at the last sampling point. Thus, the giant trevally can be categorized between the two categories, i.e., it forms Hx at significant levels, but only after the fish has passed its shelf life, while Ino is present at high levels even during the late stage of spoilage.

Cobia showed a gradual decrease in IMP down to negligible levels, fluctuating Ino levels and a slow/gradual increase in Hx, reaching intermediate concentrations. Similarly, milkfish showed a pattern that fell between the two basic categories, i.e., it gradually formed Hx at significant concentrations, but Ino also remained at high levels. The shelf life of the species was indicated by the significant (*p* < 0.05) increase in Ino on day 8, while Hx was mainly formed long after the fish had passed the acceptability limit. The coral trout can also be grouped into the intermediate category (between Hx-forming and non-forming species), but more toward the non-forming ones; the rapid increase (*p* < 0.05) in Ino on day 8 also indicated the end of its shelf life. Hx was formed slowly and increased gradually, but it remained at relatively low levels even after the fish had surpassed its shelf life. The existence of different kinetics in IMP breakdown and the variability in the formation of Ino or Hx as final products in different fish species have been previously reviewed [24], but none of the herein-studied species were included, and therefore, this is an important new finding.

**Figure 4:**
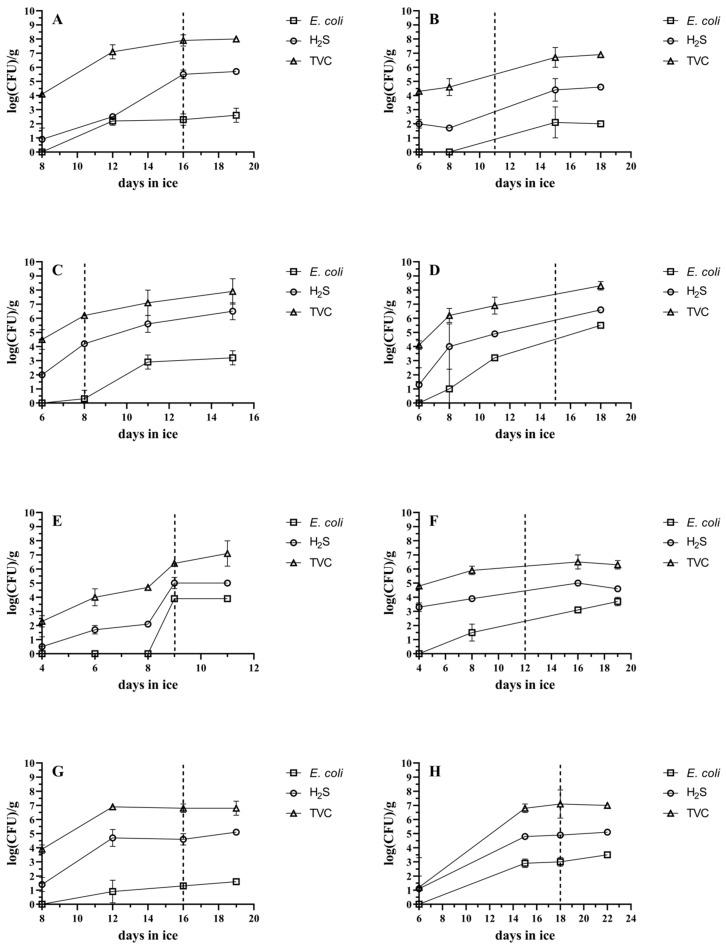
Microbial spoilage of (**A**) barramundi, (**B**) cobia, (**C**) coral trout, (**D**) giant trevally, (**E**) milkfish, (**F**) mangrove red snapper, (**G**) snubnose pompano and (**H**) sobaity bream during ice storage. TVC (△), H2S-producing bacteria (◯) and *E. coli* (◻). The dotted line represents the day that the acceptability limit was reached (end of shelf life). The bars represent the standard deviations (n = 3).

The microbial load in the muscle tissue of the studied species under ice storage is graphically presented in Figure 4. Although there is no legal safety barrier related to microbial presence in fish, a microbial load of log 6 to log 7 is generally considered the microbial threshold for fish spoilage, indicating unsatisfactory fish in good manufacturing practice [28,29]. There were cases, such as the snubnose pompano, giant trevally and barramundi, where this threshold was reached before the sensory determination of the end of shelf life. In particular, it was reached on day 12 in snubnose pompano (sensory acceptability limit: day 16), in giant trevally on day 11 (sensory acceptability limit: day 15) and in barramundi on day 12 (sensory acceptability limit: day 16). In a previous study, where a similar pompano species (*Pompano falcatus*) was stored under refrigeration, a shelf life of 18 days coincided with a total microbial load of log 6 [30]. *E. coli* is not normally part of fish’s microbiota and is considered a contaminant [31,32]. The enterobacteria load that was measured in a shelf life study of ice-stored cobia [9] was much higher after 15 days of storage than our *E. coli* measurements in cobia, but this may well be due to contamination of the stored fish, since the rest of the microbial population was similar to our findings.

## 4. Conclusions

The spoilage patterns of eight important wild and farmed Saudi Arabia-produced fish species were studied. Since there are scarce results on the freshness and shelf lives of the studied species when they are stored in ice, these results may serve as a valuable tool for freshness control and quality assurance during commercialization in local Saudi Arabian markets and the rest of the world. Our study showed that wild-caught species (cobia, coral trout, giant trevally, milkfish and mangrove red snapper) had shorter shelf lives than farmed ones (barramundi and sobaity bream), based on our evaluations using the species-specific QIM schemes. The ATP breakdown product concentrations and the K-value schemes were strictly dependent on the species, and the values at the point of rejection did not relate to whether the fish were a wild-caught or farmed species. Some species, such as the mangrove red snapper and barramundi, accumulated hypoxanthine as a final ATP product, while others, such as sobaity bream, formed inosine. The total microbial loads were within a range of log 6–8 at the end of the fish’s shelf life and were also independent of whether the fish was of farmed or wild origin.

## Figures and Tables

**Table 1:** The studied fish species, their origin and/or fishing methods and their average weights.

Common Name	Scientific Name	Origin/Fishing Method	Weight of the Studied Fish (Average in g)
Barramundi (Asian seabass)	*Lates calcarifer*	Farmed	1500
Cobia	*Rachycentron canadum*	Wild/hand line	6500
Coral trout	*Plectropomus leopardus*	Wild/hand line	920
Giant trevally	*Caranx ignobilis*	Wild/hand line	2560
Milkfish	*Chanos chanos*	Wild/surface gillnet	400
Mangrove red snapper	*Lutjanus argentimaculatus*	Wild/hand line	3200
Snubnose pompano	*Trachinotus blochii*	Farmed	1200
Sobaity bream	*Sparidentex hasta*	Farmed	1000

## Data Availability

The original contributions presented in this study are included in the article/Supplementary Material. Further inquiries can be directed to the corresponding author.

## Supplementary Materials

The following supporting information can be downloaded at https://www.mdpi.com/article/10.3390/foods14040690/s1. Tables S1–S8: Supplementary-meterial QIM schemes; Figure S1: baramundi; Figure S2: cobia; Figure S3: coral trout; Figure S4: giant trevaly; Figure S5: milkfish; Figure S6: mangrove red snapper; Figure S7: snubnose pompano; Figure S8: sobaity bream.
