# Supplementary material for: Freshness and Spoilage Patterns of Wild and Farmed Tropical Fish Species with Major Commercial Importance Originating from Saudi Arabian Waters
Source: Foods. 2025 Feb 17;14(4):690. doi: 10.3390/foods14040690 (PMC11853878; doi:10.3390/foods14040690)
Supplement: Supplementary file 1 [file foods-14-00690-s001.zip › Supplementary-material-QIM-schemes.pdf]

Table S1 QIM scheme for barramundi

Feature		Description	Score
Appearance	Slime	Excess of clear-transparent	0
		Reduction of slime	1
		Thick milky slime (yellow/grey/brown)	2
	Scales	Firmly attached	0
		Easy to detach	1
Belly	Appearance	Firm	0
		Soften or burst	1
Flesh		Firm/very elastic	0
		Hard (rigor)	1
		Soften	2
Eyes	Clarity	Clear-transparent	0
		Slightly opaque	1
		Completely opaque	2
	Pupil	White transparent	0
		Cloudy white (opaque)	1
	Shape	Convex	0

		Flat	1
		Sunken/concave or popped out	2
Gills	Colour	Bright/dark red	0
		Light red/red with pink edges	1
		Brown/purple/pink (discolored)	2
	Mucus	Thin transparent	0
		Thick sticky/clotted/white or brown	1
	Smell	Fresh sea/seaweed	0
		Neutral	1
		Bad (swamp/rubbish)	2
	**TOTAL**		**16**

Table S2 QIM scheme for cobia

Feature		Description	Score
Appearance	Skin	Very bright	0
		Loss of brightness	1
		Dull	2
	Slime	Clear-transparent	0
		Absence (dry) or milky slime	1
Belly	Appearance	Firm	0
		Soften or burst	1
Flesh	Elasticity	Elastic	0
		Marked by pressure	1
	Odour	Fresh	0
		Neutral	1
		Fishy	2
		Off odors	3
Eyes	Clarity	Clear-translucent	0
		Slightly opaque	1
		Opaque/bloody	2
	Pupil	Black	0

Feature		Description	Score
		Cloudy	1
	Shape	Convex	0
		Flat	1
		Sunken/concave	2
Gills	Colour	Bright/dark red	0
		Pale red/pink	1
		Brownish/purple/discolored	2
	Mucus	Thin transparent	0
		Sticky/clotted/white	1
	Smell	Fresh/seaweed	0
		Neutral/metallic	1
		Fishy	2
		Off odors/sulphuric/fecal	3
**TOTAL**			**20**

Table S3 QIM scheme for coral trout

Feature		Description	Score
Appearance	Skin Color	Uniform bright red	0
		Loss of brightness (dull) yellow or white discoloration (aerial: gill cover, tail, ventral)	1
		Extensive white or yellow discoloration	2
	Spots	Bright (black or blue) - well defined borders	0
		Dull (loss of intensity)/fading	1
	Slime	Clear-transparent	0
		Absence (dry) or thick milky slime	1
Belly	Appearance	firm	0
		Soften/sunken or burst	1
Anus		Closed	0
		Open	1
Flesh	Elasticity	Soft	0
		Hard	1

Feature		Description	Score
Eyes	Clarity	Clear-translucent	0
		Slightly opaque	1
		Opaque	2
	Pupil	Black	0
		Cloudy	1
	Shape	Convex	0
		Flat	1
		Sunken/concave or popped out	2
Gills	Colour	Bright/dark red	0
		Red brown/pink	1
		Beige/light brown	2
	Mucus	Thin transparent	0
		Thick sticky/clotted/brown	1
	Smell	Fresh sea/seaweed	0
		Neutral/subtle/medicinal	1
		Bad (garbage/sulphuric/fecal)	2
**TOTAL**			**17**

Table S4 QIM scheme for giant trevally

Feature		Description	Score
Appearance	Skin	Bright	0
		Dull	1
	Slime	Clear-transparent	0
		Absence (dry) or thick milky slime	1
	Scales	Firmly attached	0
		Easy to detach or loss	1
Belly	Appearance	Firm	0
		Soften or burst	1
	Color	Normal	0
		Yellow discoloration	1
Anus		Closed	0
		Open	1
Flesh	Elasticity	Elastic	0
		Hard	1
		Unelastic/marked by pressure	2
	Odour	Fresh	0

		Neutral	1
		Fishy	2
Eyes	Clarity	Clear-translucent	0
		Slightly opaque	1
		Opaque/bloody	2
	Pupil	Black	0
		Cloudy	1
	Shape	Convex	0
		Flat	1
		Sunken/concave or popped out	2
Gills	Colour	Bright/dark red	0
		Red with pink edges	1
		Pale red/pink	2
	Mucus	Thin transparent	0
		Thick sticky/clotted/white	1
	Smell	Fresh sea/seaweed	0
		Neutral	1
		Insipid (metallic/ oxidation)	2

	Bad (sulphuric/fecal)	3
**TOTAL**		**21**

Table S5 QIM scheme for milkfish

Feature		Description	Score
Appearance	Skin	Very shiny/iridescent	0
		Shiny	1
	Slime	Little/clear-transparent	0
		Increased/thick slime	1
	Scales	Attached	0
		Loose or some visible loss of scales	1
		Extensive loss of scales	2
Belly	Appearance	Firm	0
		Soft	1
		Decomposing/seizures	2
	Color	Normal	0
		Black discoloration (spotted)	1
		Extensive black discoloration	2
Anus		Closed	0
		Open	1
Flesh	Elasticity	Elastic (bounces back)	0
		Soft	1

Eyes	Clarity	Clear-translucent	0
		Slightly opaque	1
		Opaque	2
	Pupil	Black	0
		Cloudy/white	1
	Shape	Convex	0
		Flat	1
		Sunken/concave	2
Gills	Colour	Dark red/dark brown	0
		Red/pink	1
		Beige/purple	2
	Mucus	Thin transparent	0
		Thick sticky/clotted/white	1
	Smell	Fresh sea/seaweed	0
		Neutral/metallic	1
		Stagnant water	2
		Bad (sulphuric/fecal)	3
**TOTAL**			**21**

Table S6 QIM scheme for mangrove red snapper

Feature		Description	Score
Appearance	Slime	Little/transparent	0
		Absence (dry) or thick milky slime	1
	Scales	Firmly attached	0
		Loose scales or loss	1
	Body Color	Bright red	0
		White discoloration locally	1
		Extensive dicoloration	2
Belly	Appearance	Firm	0
		Soften or burst	1
Anus		Closed	0
		Open	1
Flesh	Elasticity	Hard/elastic	0
		Soft unelastic/marked by pressure	1
	Odour	Fresh	0
		Neutral	1
		Fishy/garbage/decomposing	2

Eyes	Clarity	Clear-translucent	0
		Slightly opaque	1
		Opaque/bloody	2
	Pupil	Black	0
		Cloudy	1
	Shape	Convex	0
		Flat	1
		Sunken/concave	2
Gills	Colour	Bright/dark red	0
		Red-brown	1
		Pink/purple	2
	Mucus	Thin transparent	0
		Thick sticky brown/clotting	1
	Smell	Fresh sea/seaweed	0
		Neutral	1
		Insipid (stagnant/metallic/oxidised blood)	2
		Bad (sulphuric/fecal/rotten)	3
**TOTAL**			**20**

Table S7 QIM scheme for snubnose pompano

	Feature	Description	Score
Appearance	Skin Color	Very irridiscent	0
		Loss of brightness	1
		Dull	2
	Slime	Clear-transparent	0
		Thick (white, brown or yellow) slime	1
Flesh	Elasticity	Very hard (rigor)/elastic	0
		hard but not elastic (pressed by finger)	1
	Odour	Fresh	0
		Neutral	1
		Harbour/garbage	2
Eyes	Clarity	Clear-translucent	0
		Slightly opaque	1
		Opaque	2
	Pupil	Black	0
		Cloudy	1
	Shape	Convex	0

		Flat	1
		Sunken/concave or popped out	2
Gills	Colour	Bright/dark red	0
		Red brown/pink	1
	Mucus	Thin transparent	0
		Thick sticky/clotted/brown	1
	Smell	Fresh sea/seaweed	0
		Neutral	1
		Insipid (metallic/medicinal/stagnant water)	2
		Bad (garbage/sulphuric/fecal)	3
	**TOTAL**		**16**

Table S8 QIM scheme for sobaity bream

Feature		Description	Score
Appearance	Colour	Bright typical	0
		Discoloration of head (patches of grey color)/belly (yellow)	1
	Slime	Clear-transparent slime	0
		Absence (dry) or thick milky slime	1
Anus		Closed	0
		Open	1
Flesh	Elasticity	Very elastic	0
		Hard/rigor	1
		Marked by pressure	2
Eyes	Clarity	Clear-transparent	0
		Slightly opaque	1
		Opaque/bloody	2
	Pupil	Black	0
		Cloudy	1
	Shape	Very convex	0

		Flat	1
		Sunken/concave	2
Gills	Colour	Dark red/bright red	0
		Red with pink edges/brown	1
		Pink/beige	2
	Mucus	Thin transparent	0
		Increased thickness/sticky	1
	Smell	Fresh sea/seaweed	0
		Neutral	1
		Insipid (metallic/ oxidation)	2
		Bad (sulphuric/ fecal/swamp/rubbish)	3
**TOTAL**			**16**
