# Supplementary figures and images for: Freshness and Spoilage Patterns of Wild and Farmed Tropical Fish Species with Major Commercial Importance Originating from Saudi Arabian Waters
Source: Foods. 2025 Feb 17;14(4):690. doi: 10.3390/foods14040690 (PMC11853878; doi:10.3390/foods14040690)

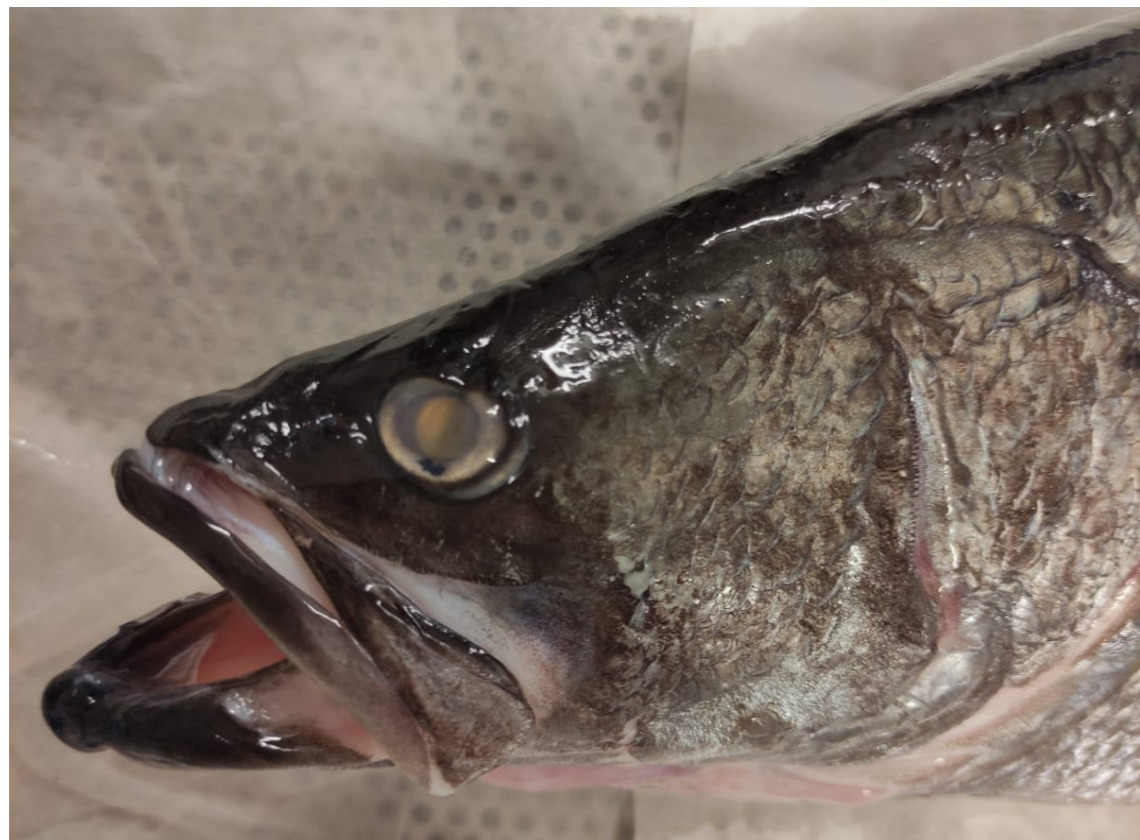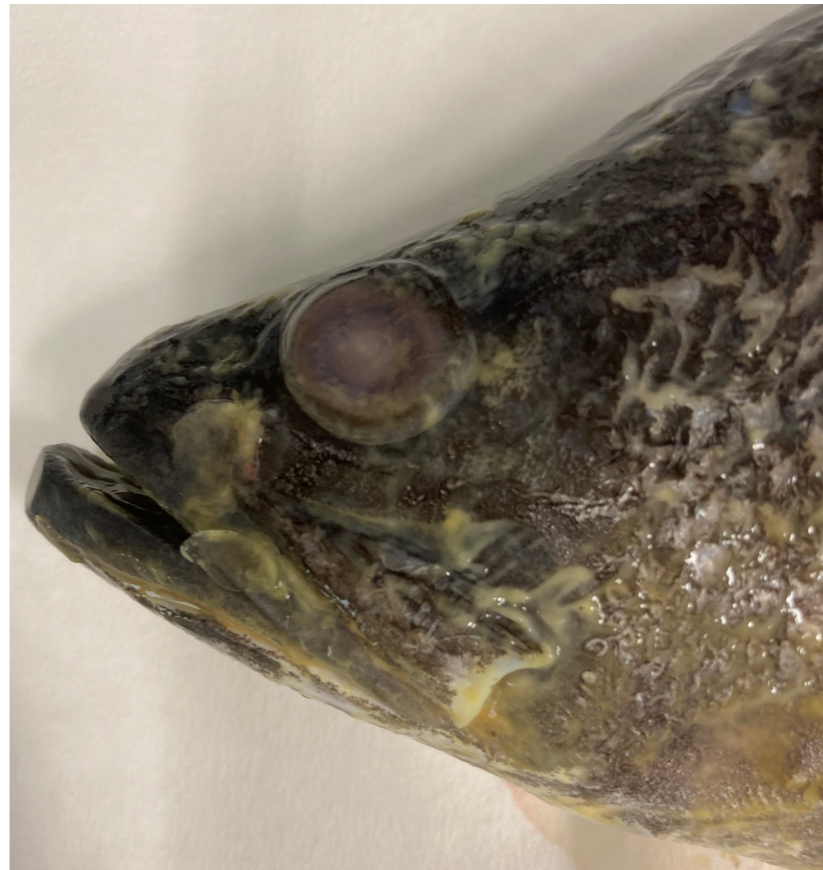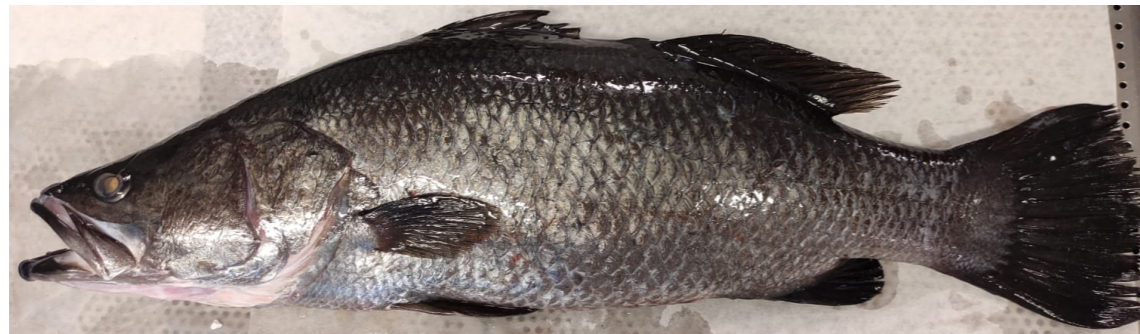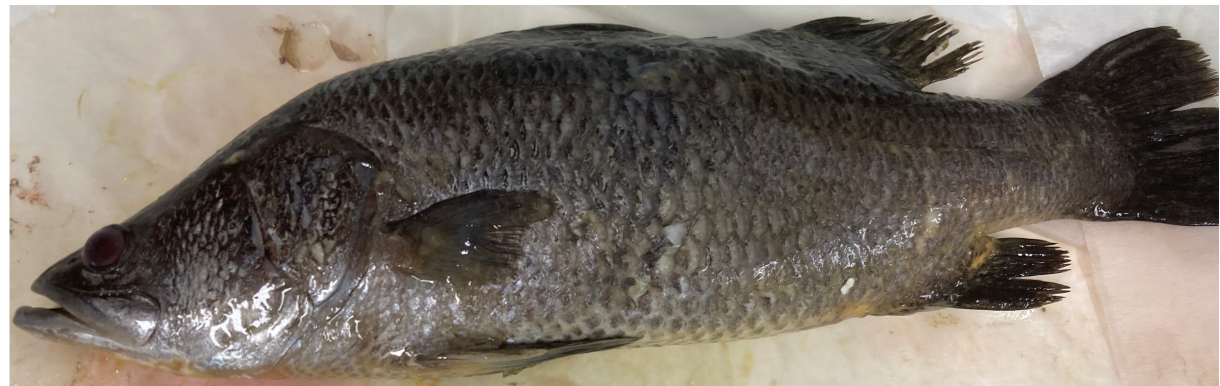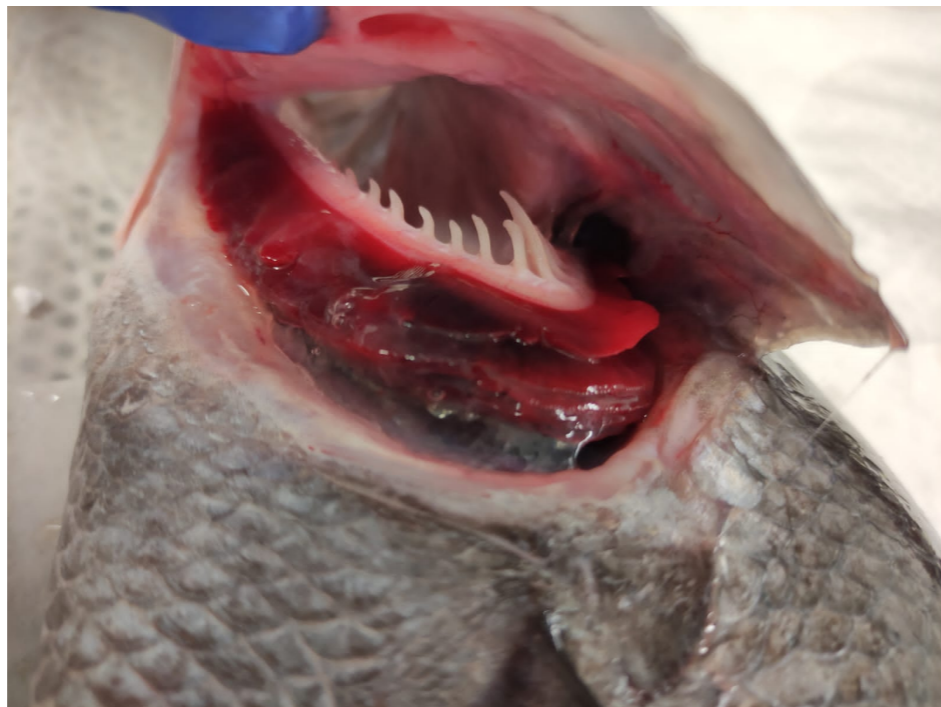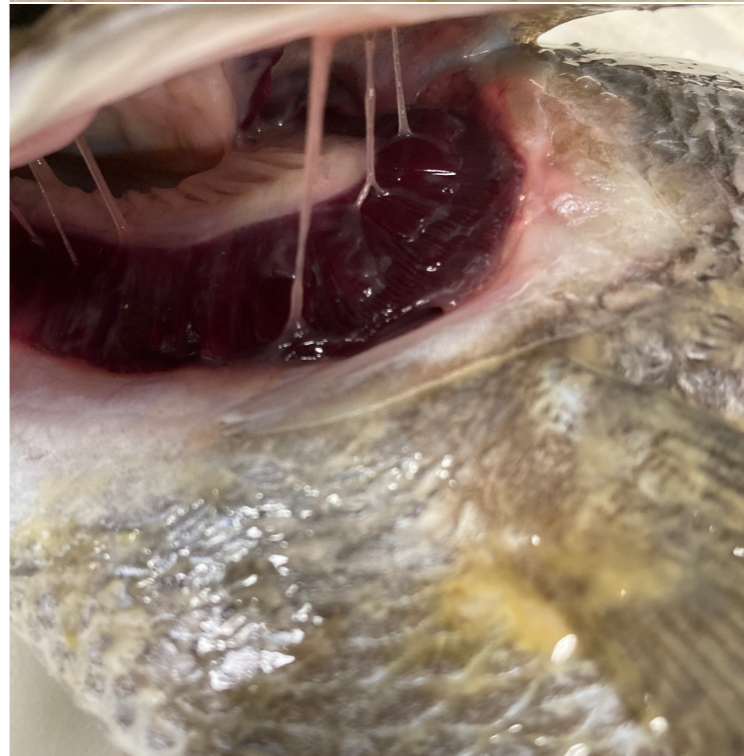

barramundi

Supplement: Supplementary file 1 [file foods-14-00690-s001.zip › Figure S1 barramundi.pdf]

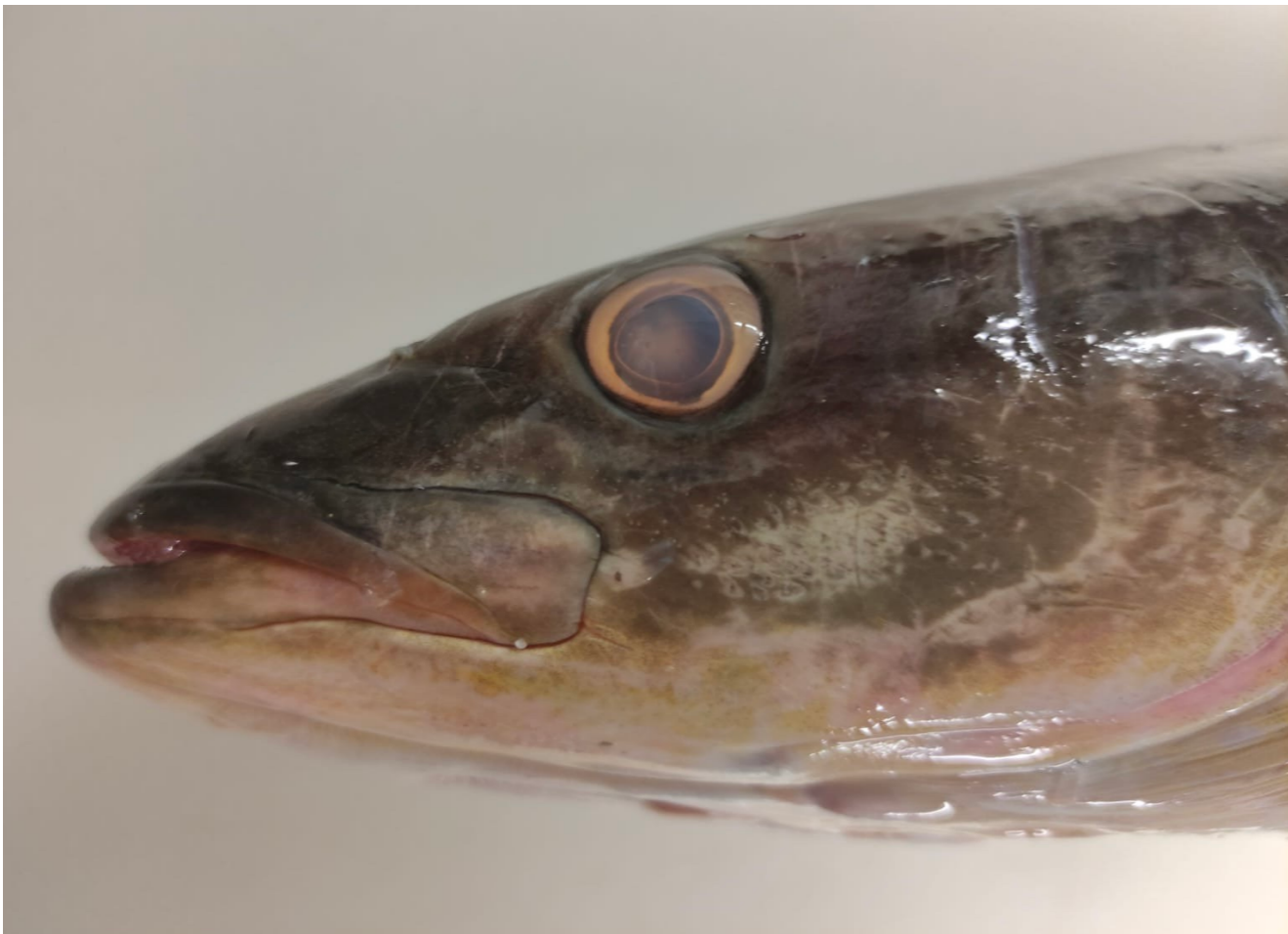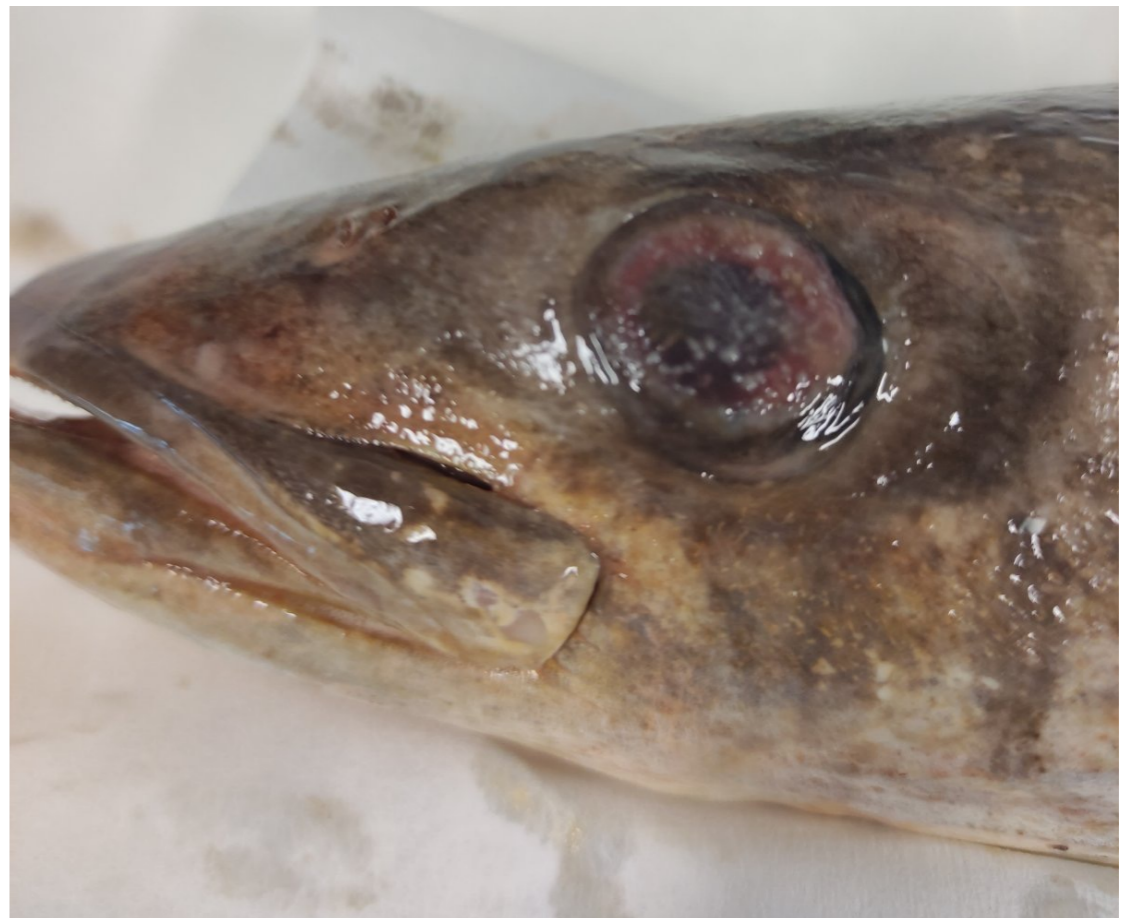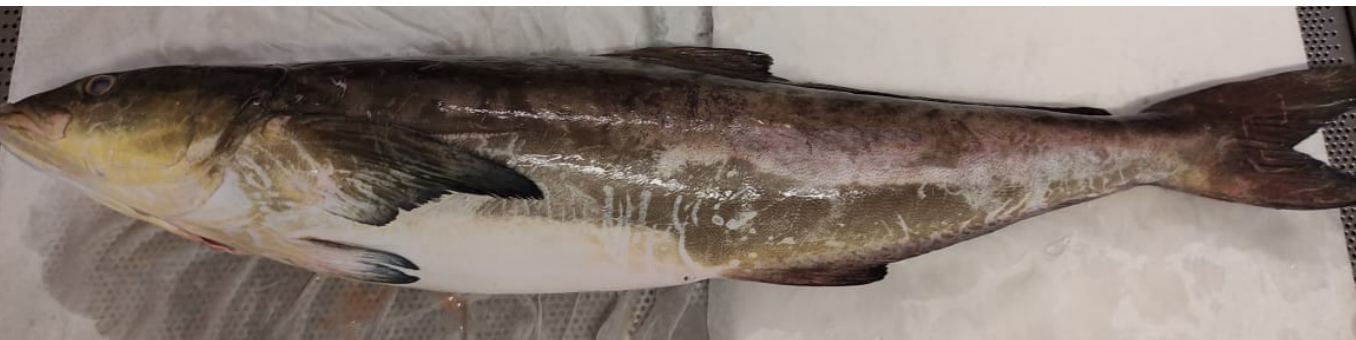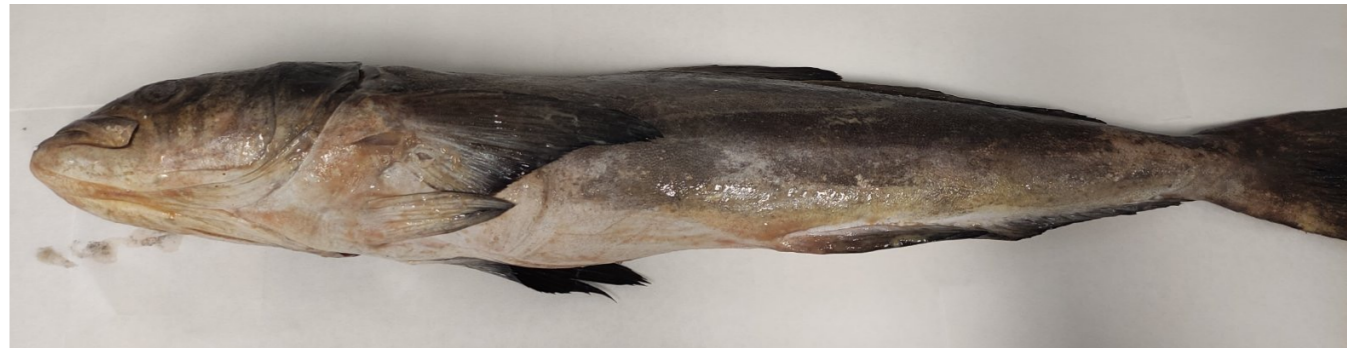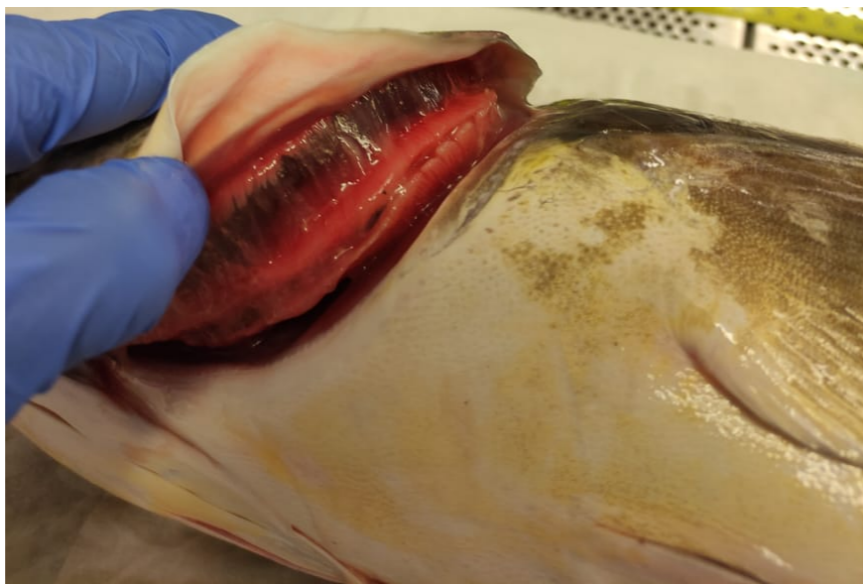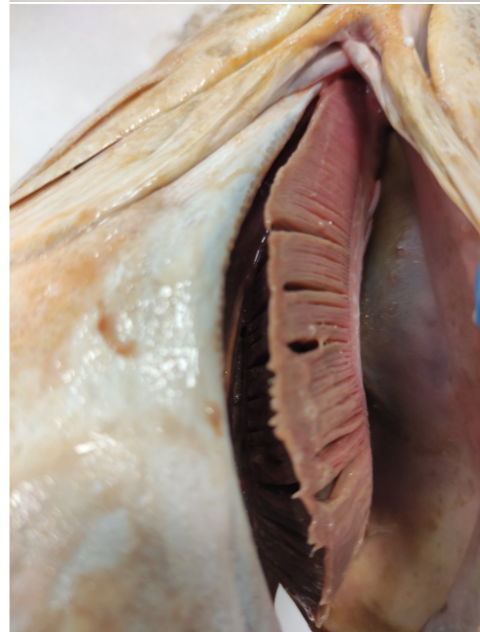

**cobia**

Supplement: Supplementary file 1 [file foods-14-00690-s001.zip › Figure S2 cobia.pdf]

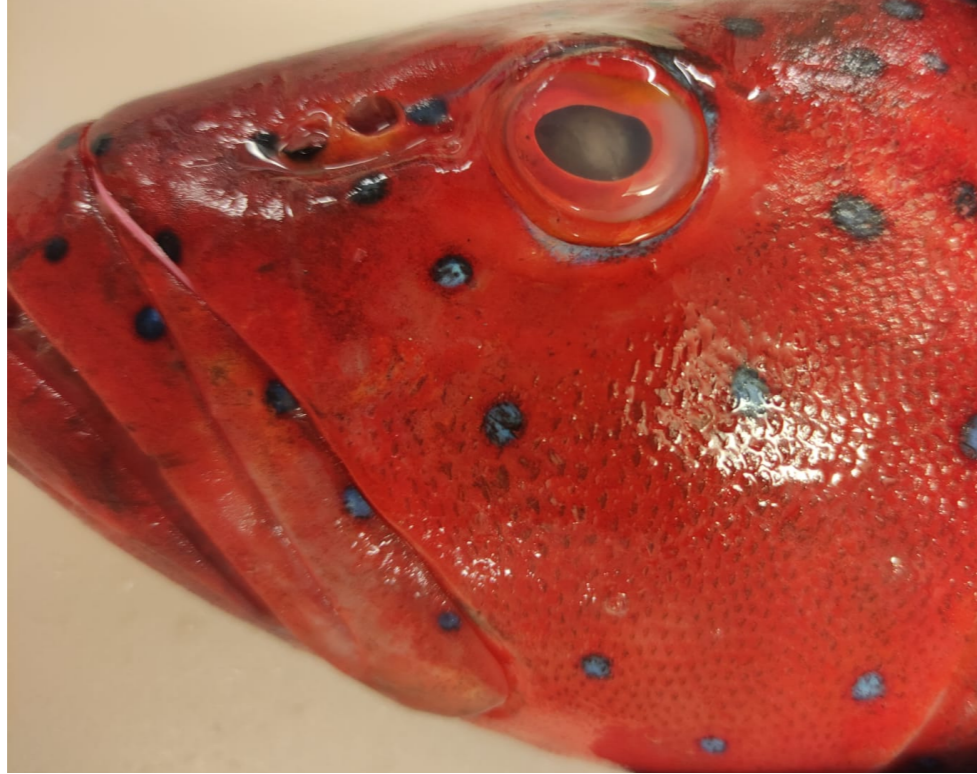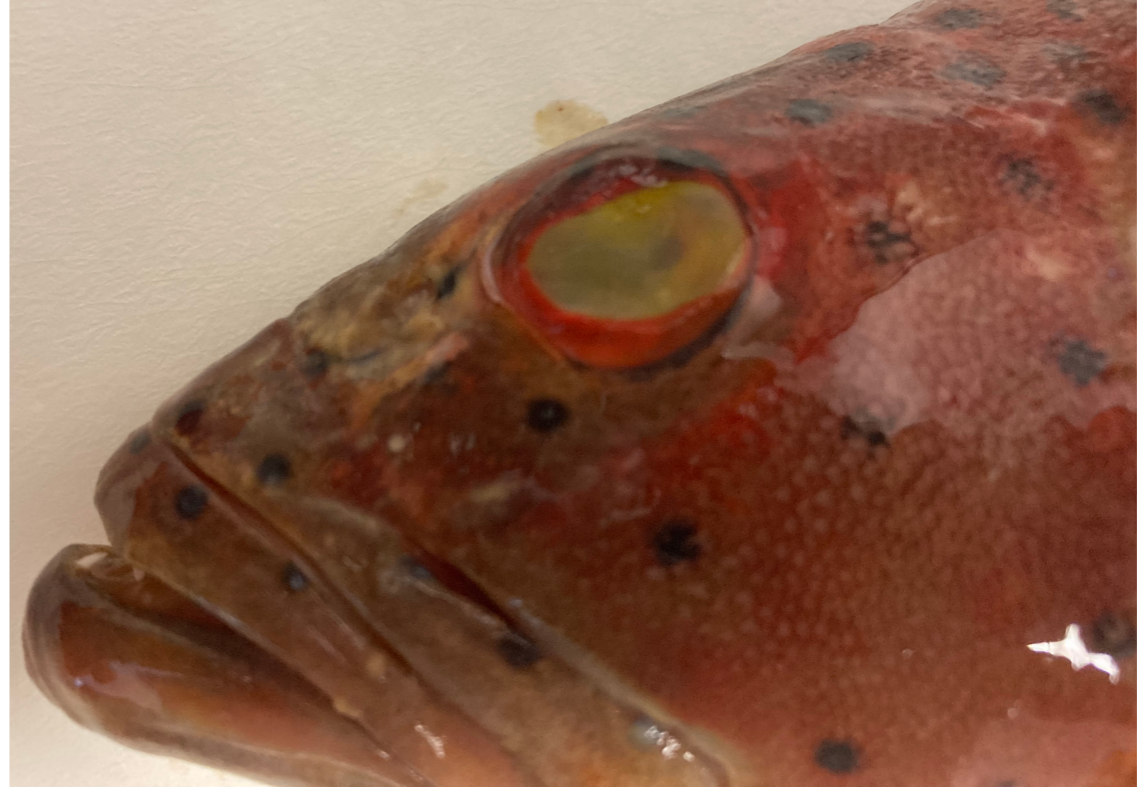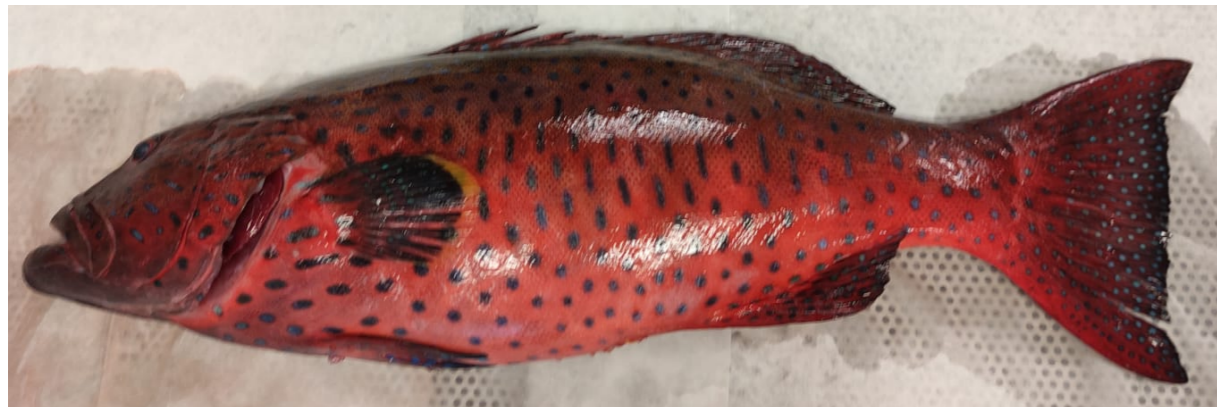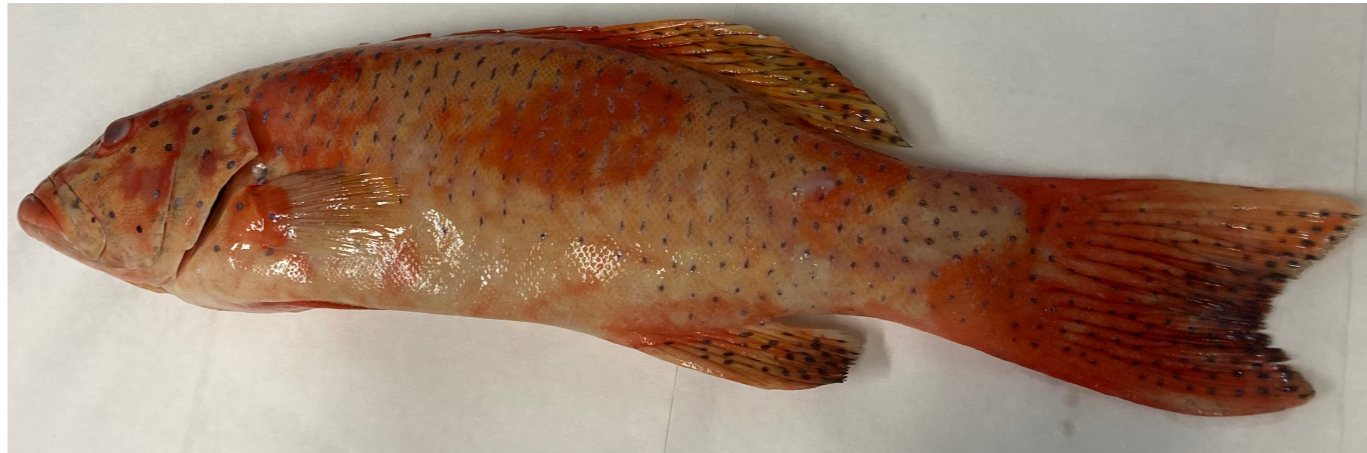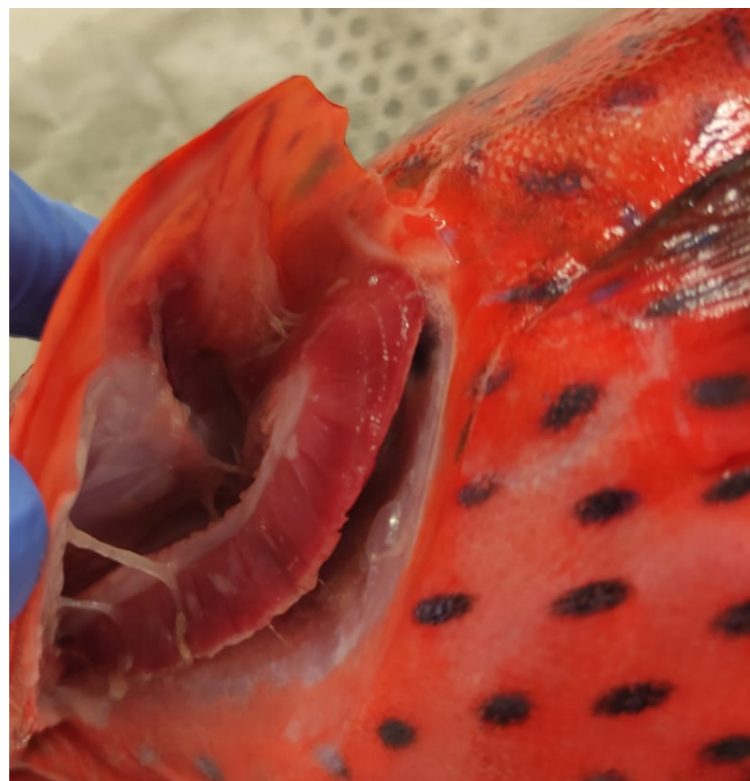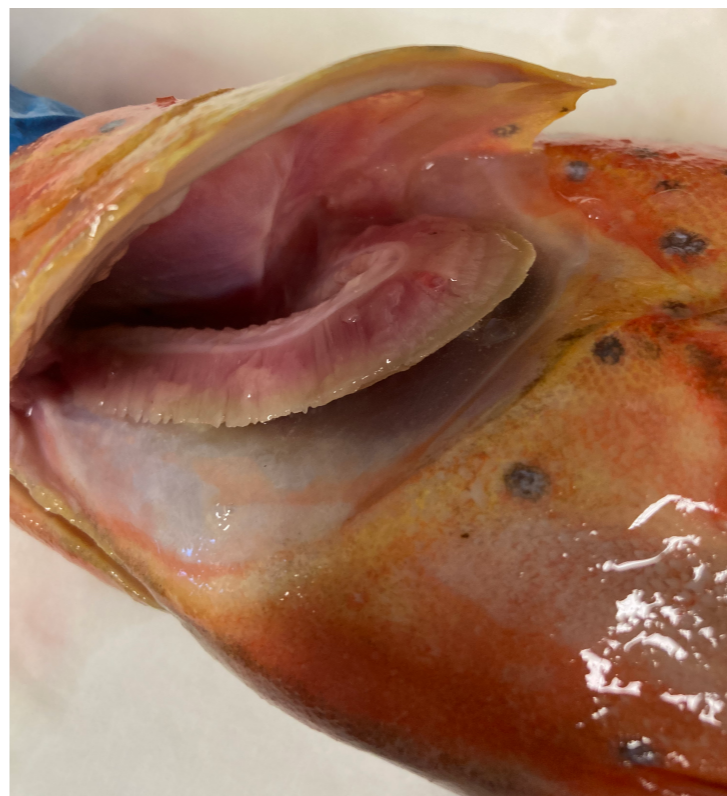

**coral trout**

Supplement: Supplementary file 1 [file foods-14-00690-s001.zip › Figure S3 coral trout.pdf]

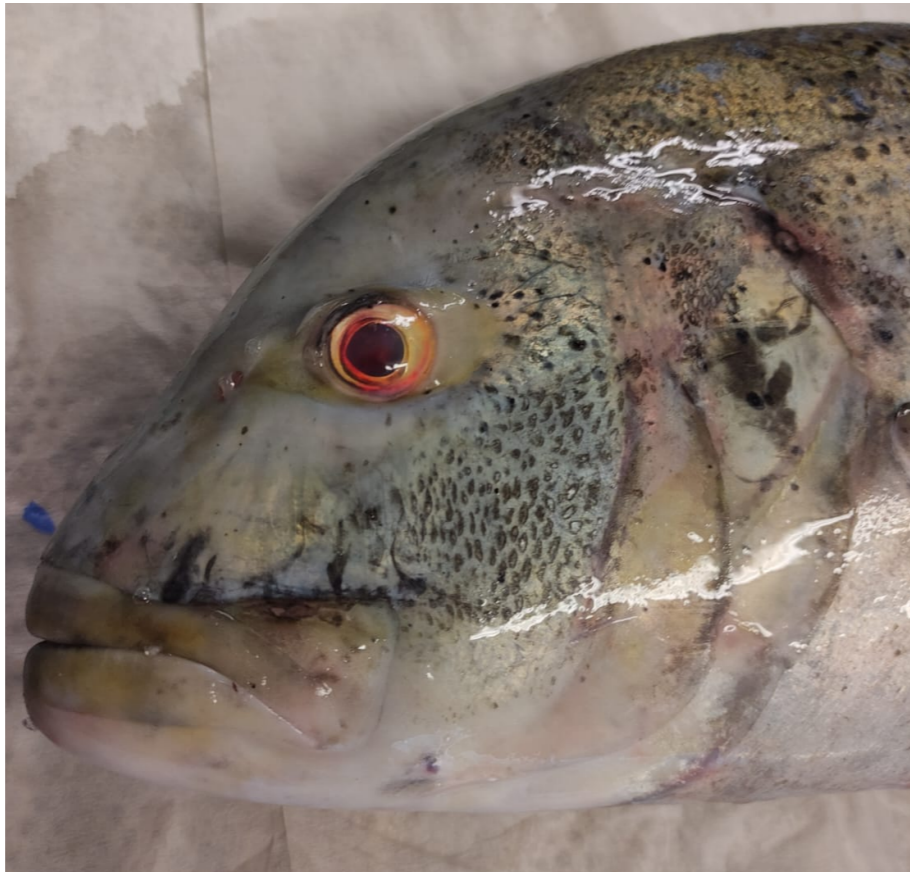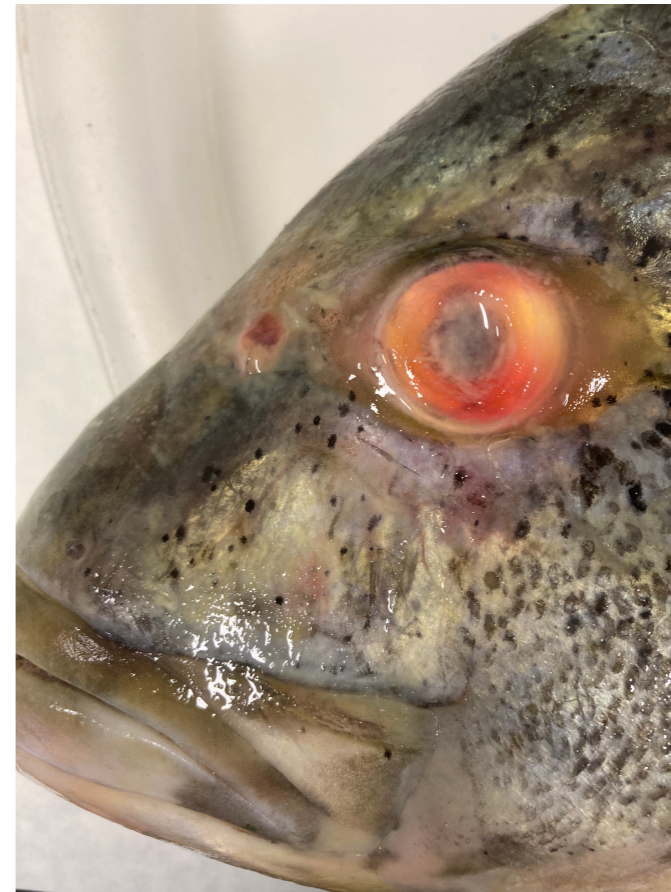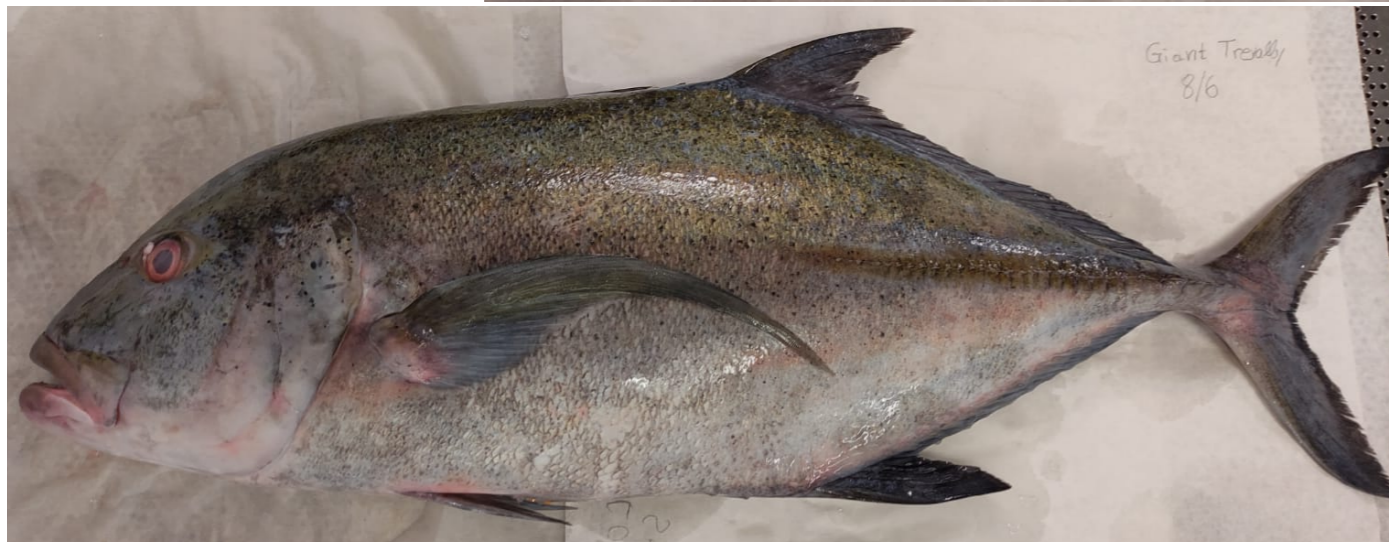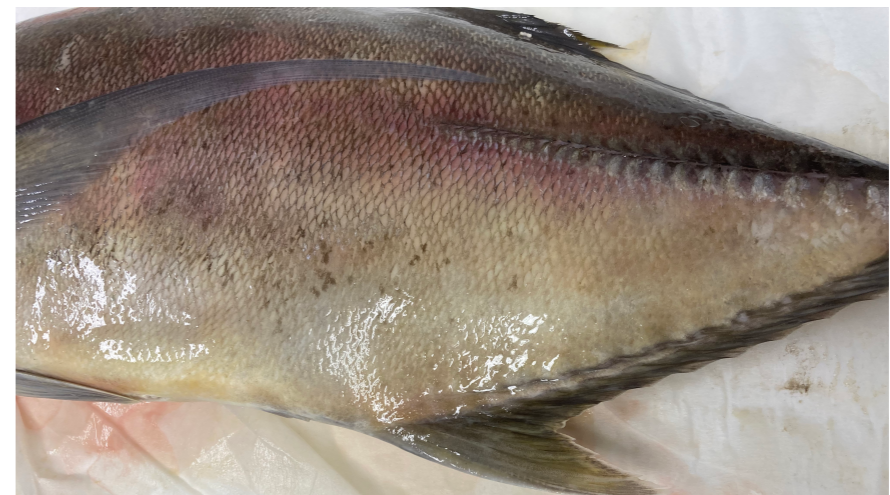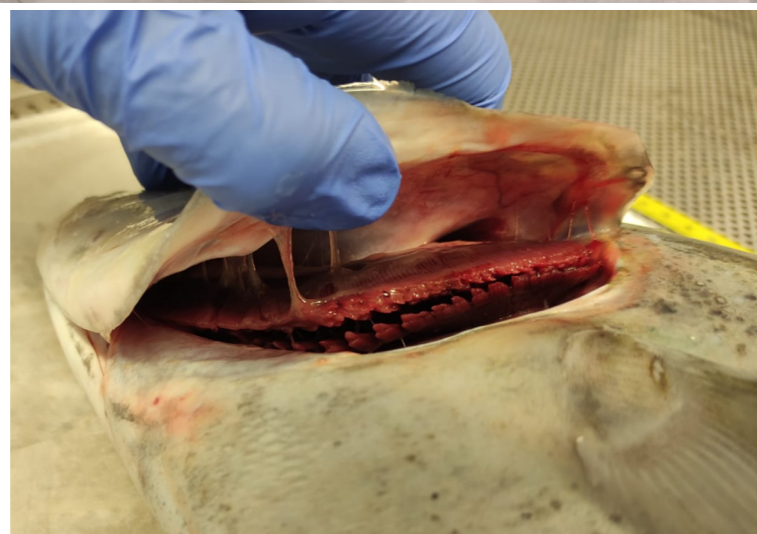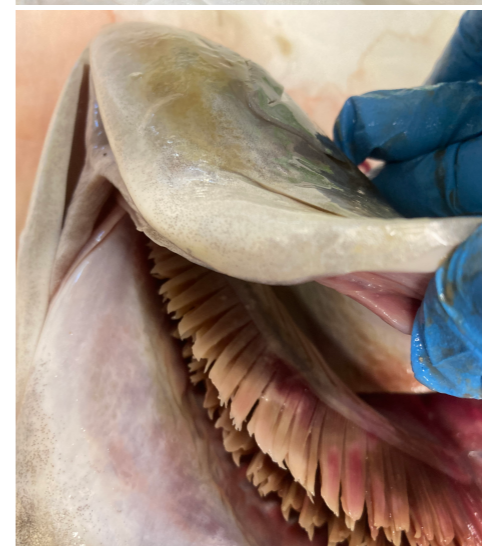

**giant trevally**

Supplement: Supplementary file 1 [file foods-14-00690-s001.zip › Figure S4 giant trevally.pdf]

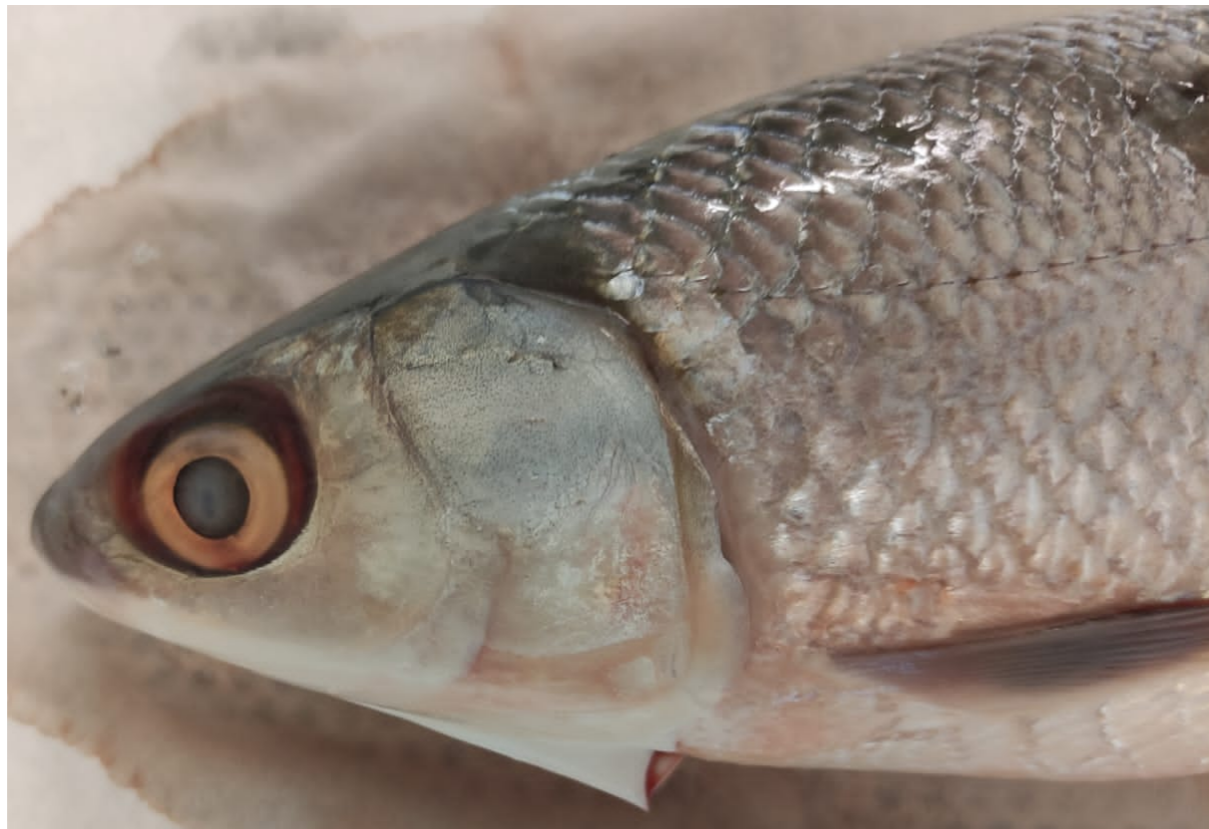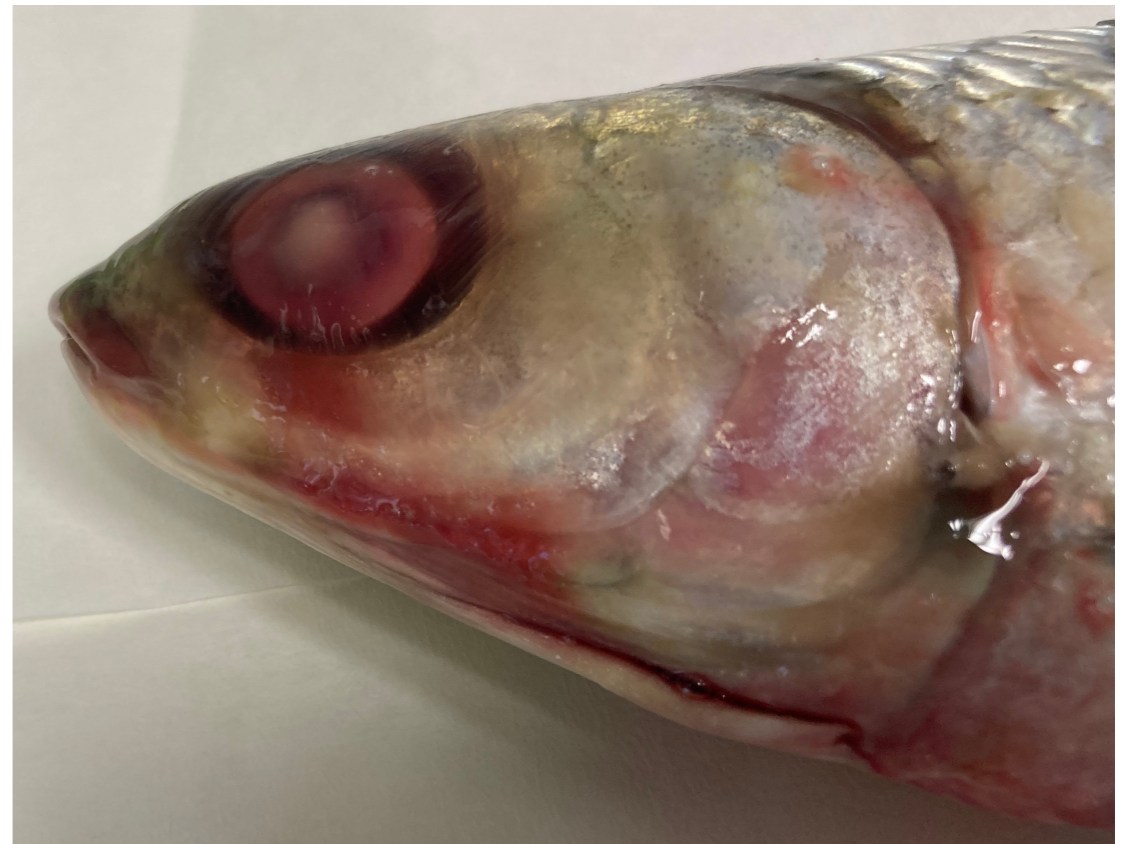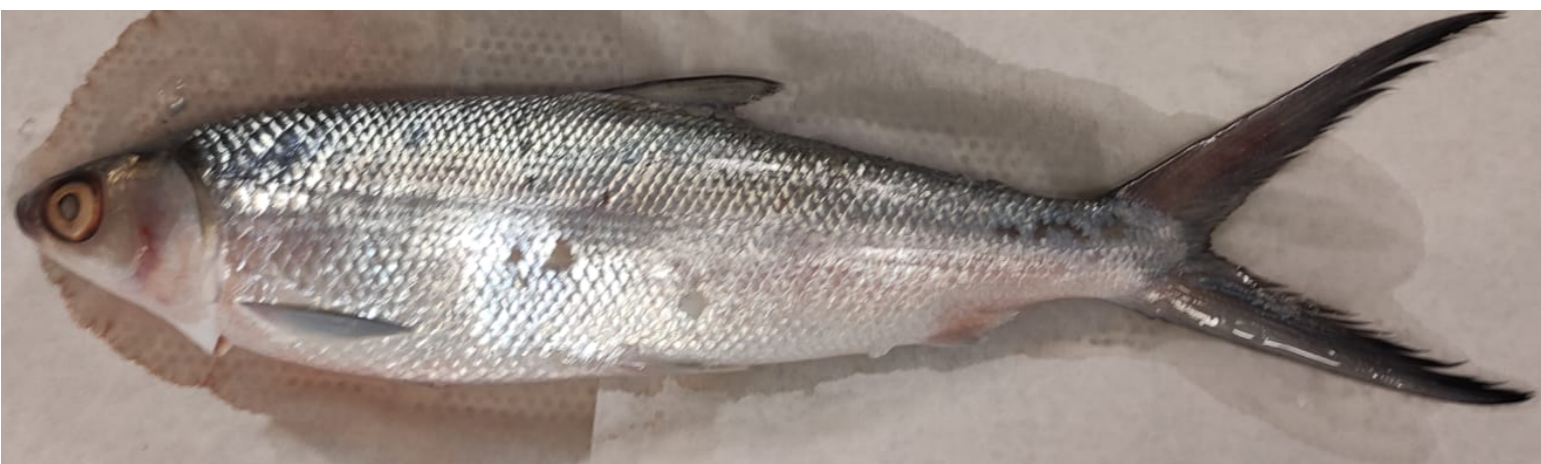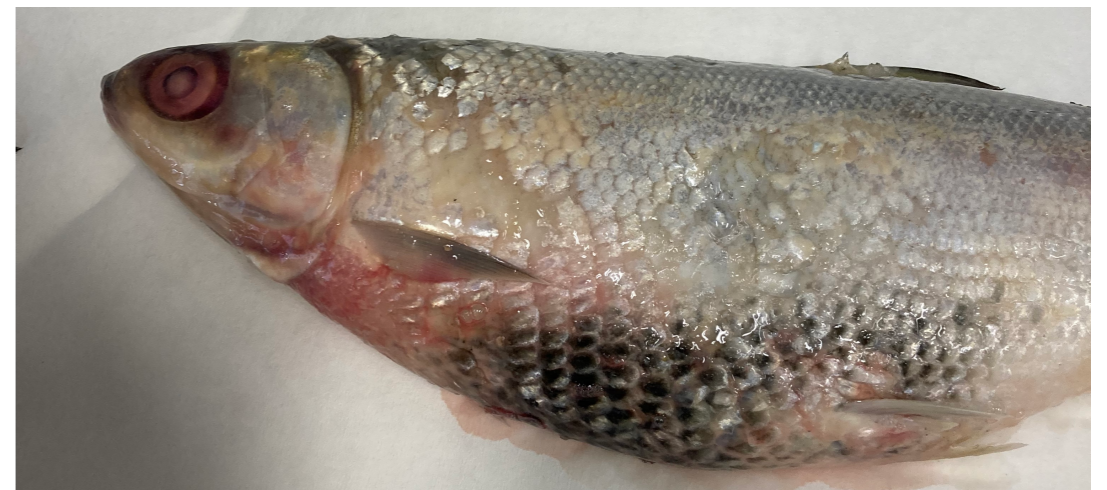

**milkfish**

Supplement: Supplementary file 1 [file foods-14-00690-s001.zip › Figure S5 milkfish.pdf]

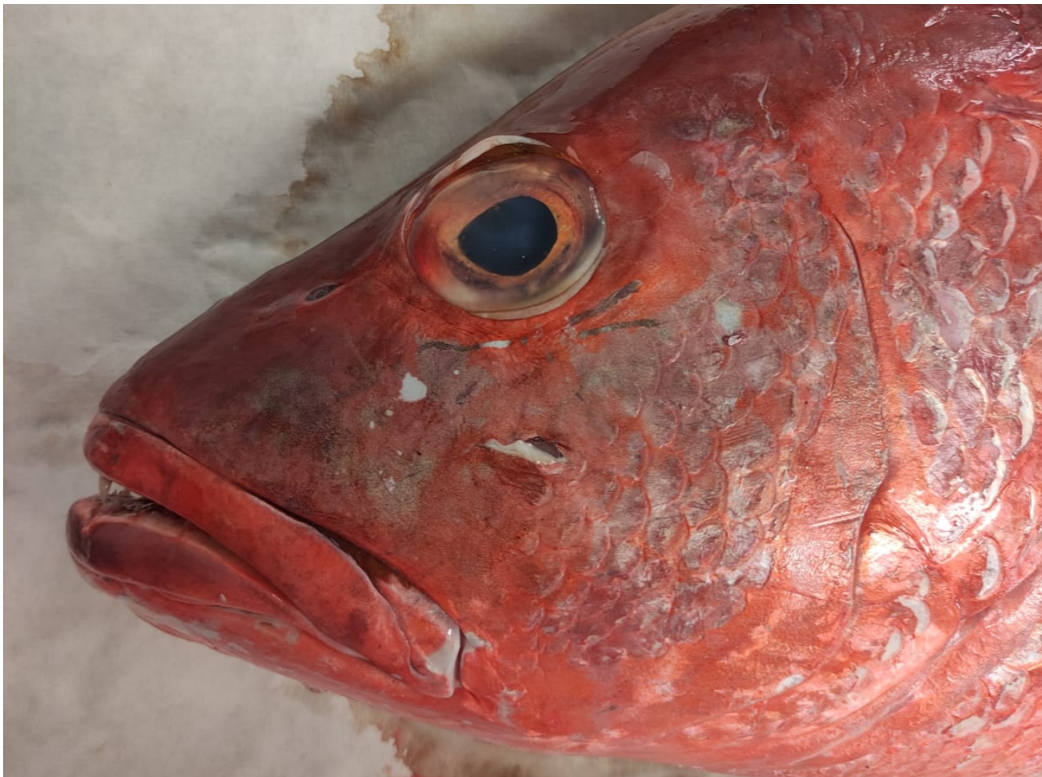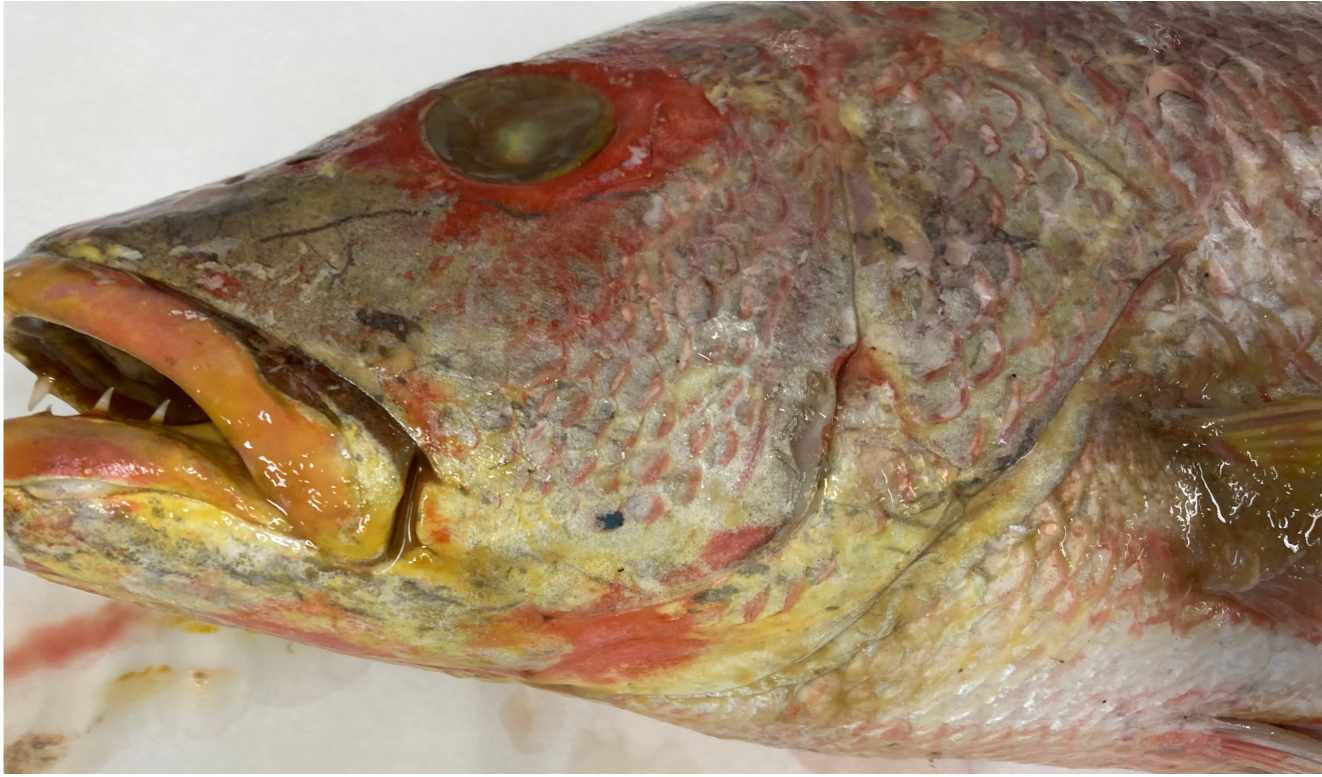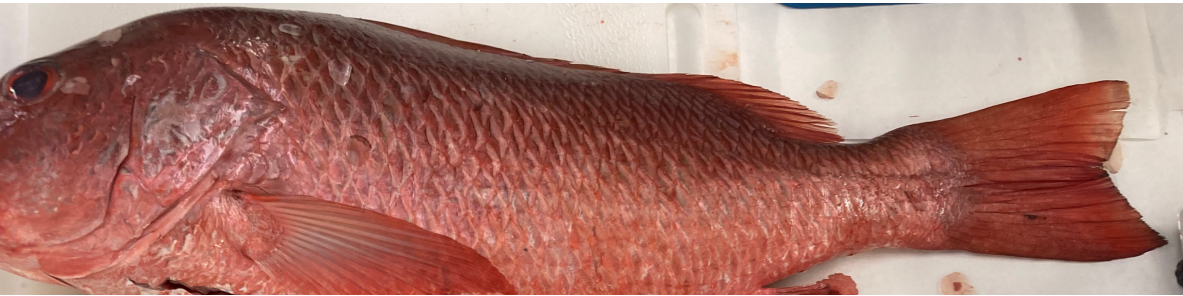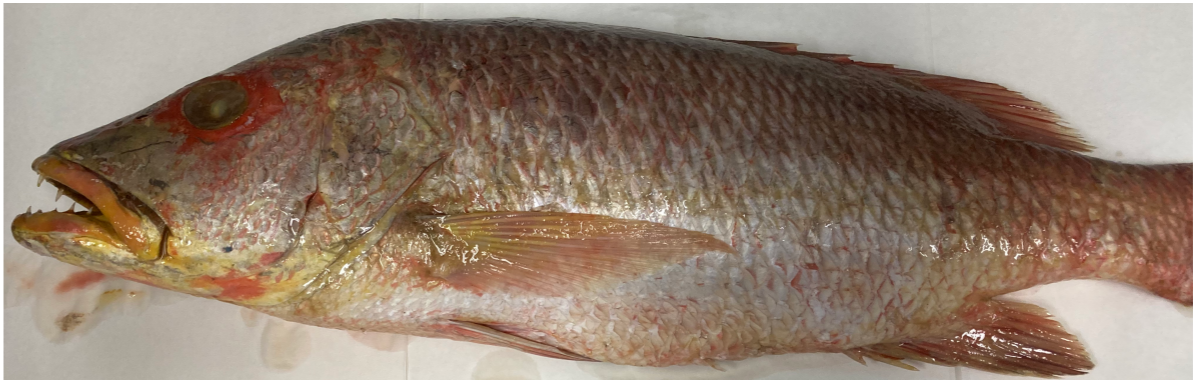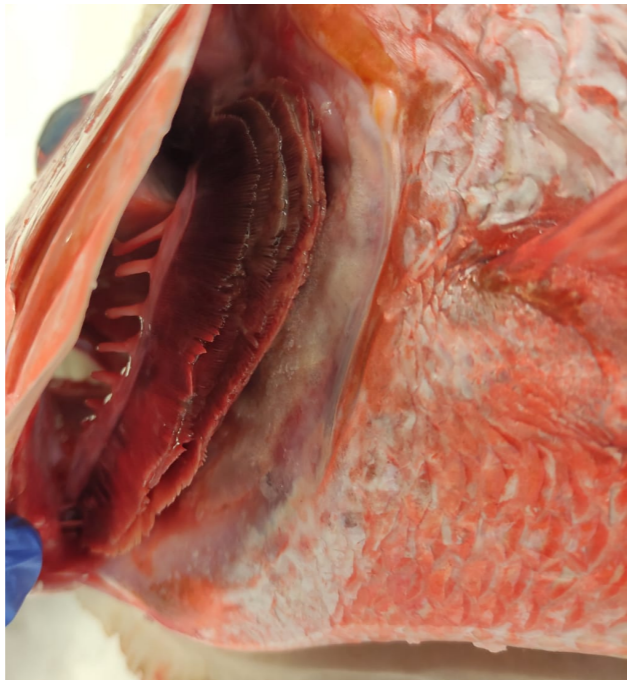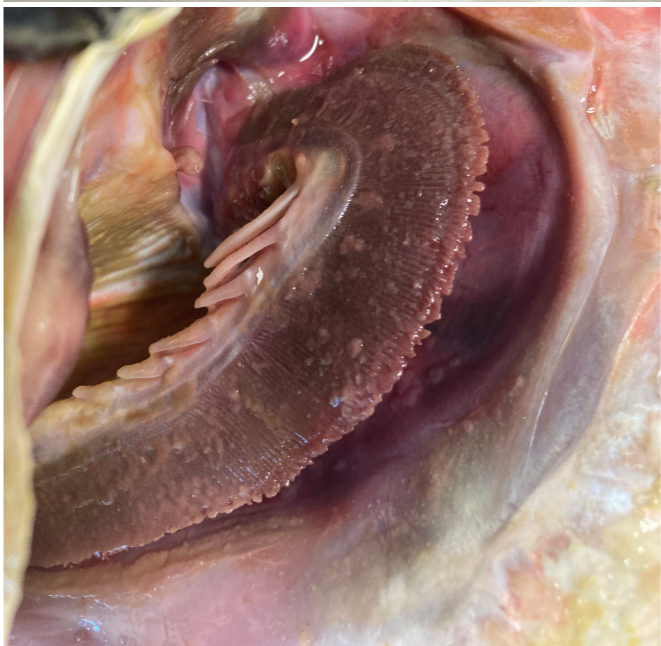

**Mangrove red snapper**

Supplement: Supplementary file 1 [file foods-14-00690-s001.zip › Figure S6 mangrove red snapper.pdf]

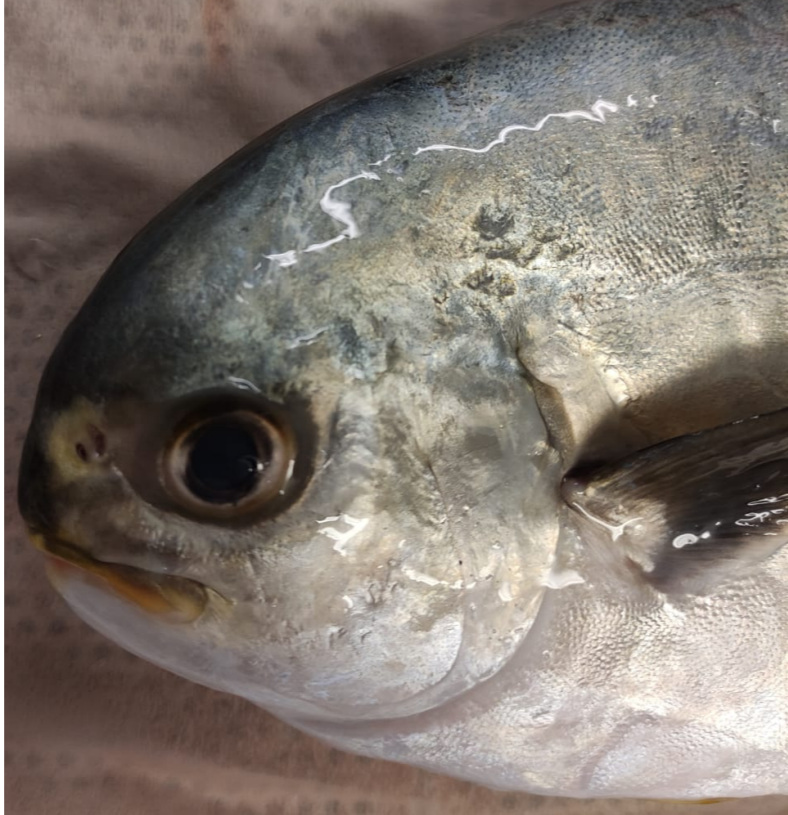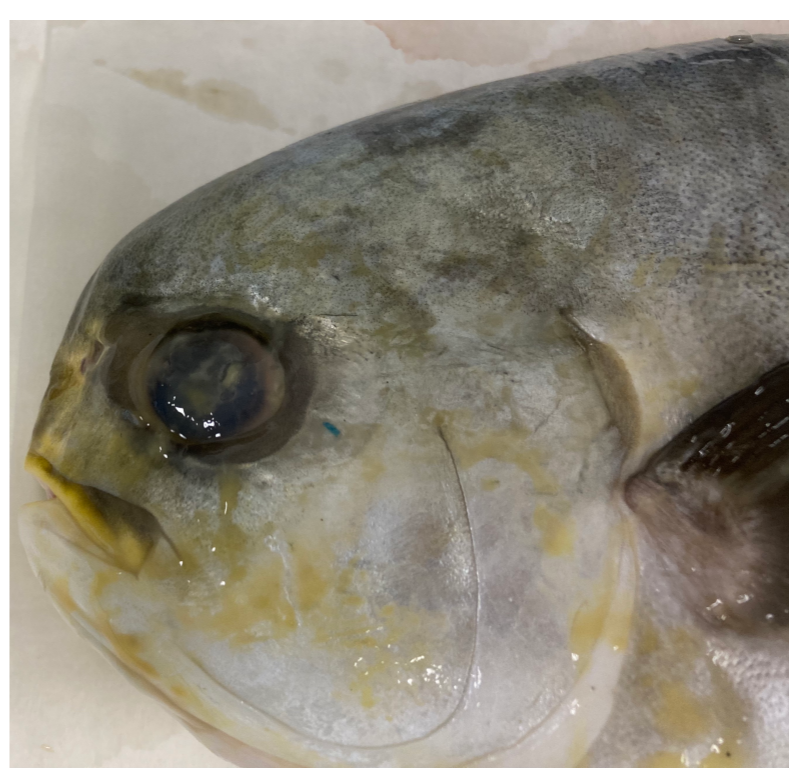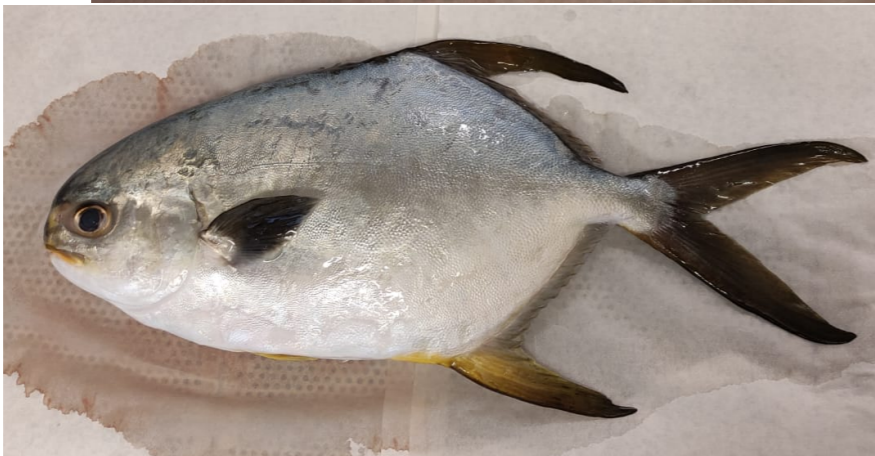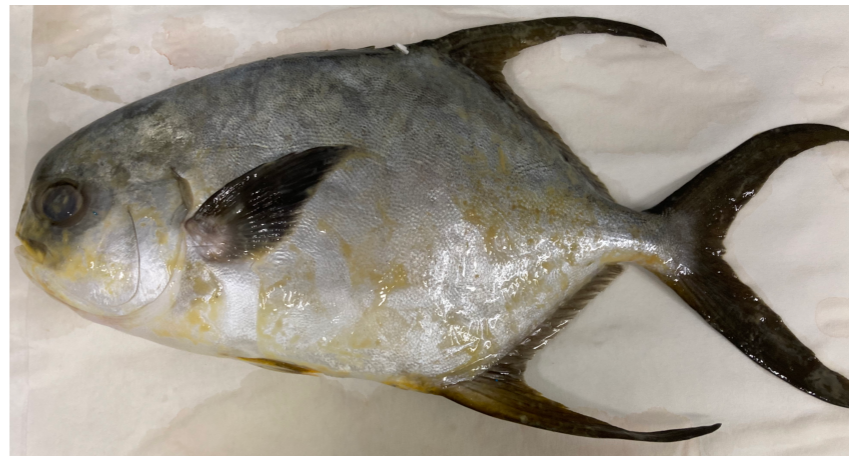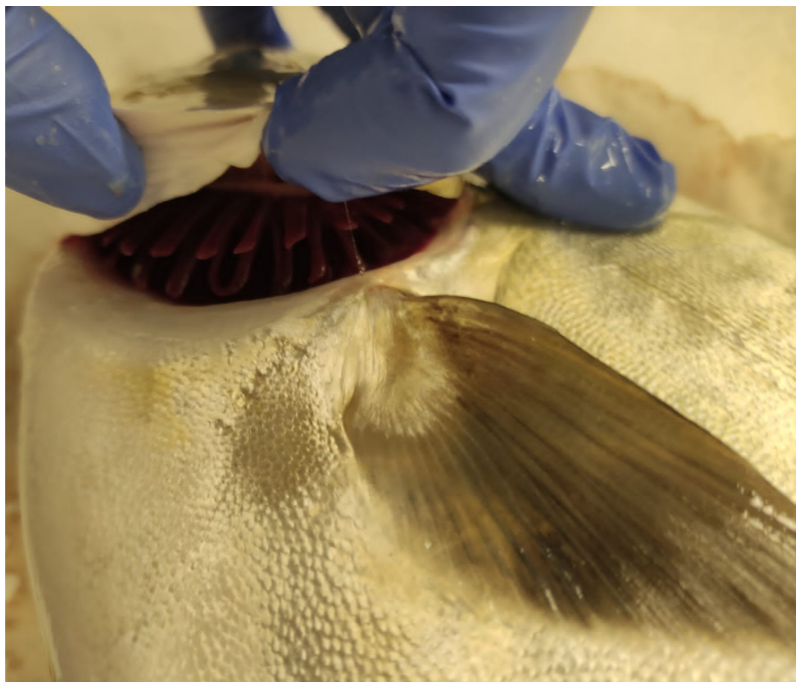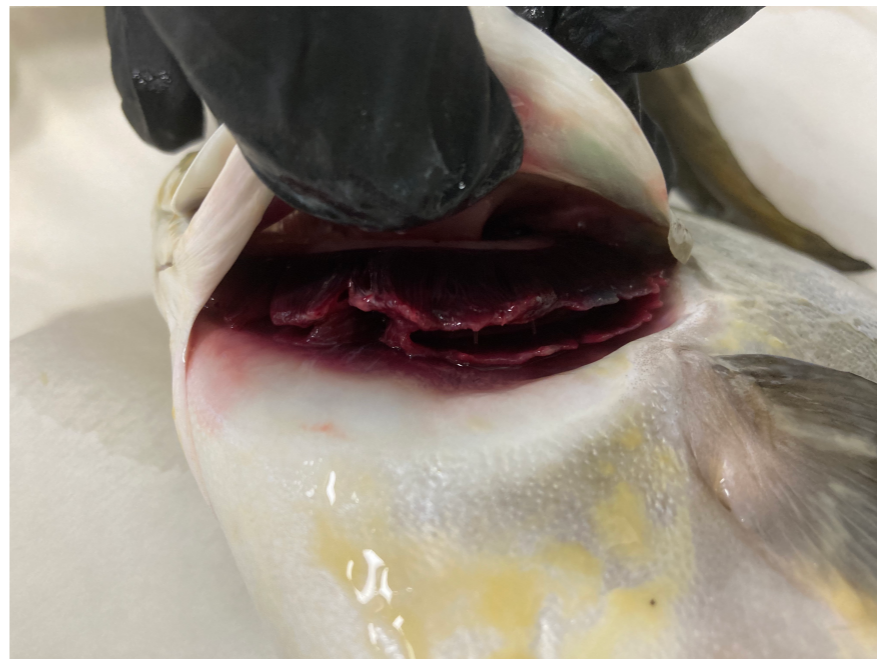

**s. pompano**

Supplement: Supplementary file 1 [file foods-14-00690-s001.zip › Figure S7 snubnose pompano.pdf]

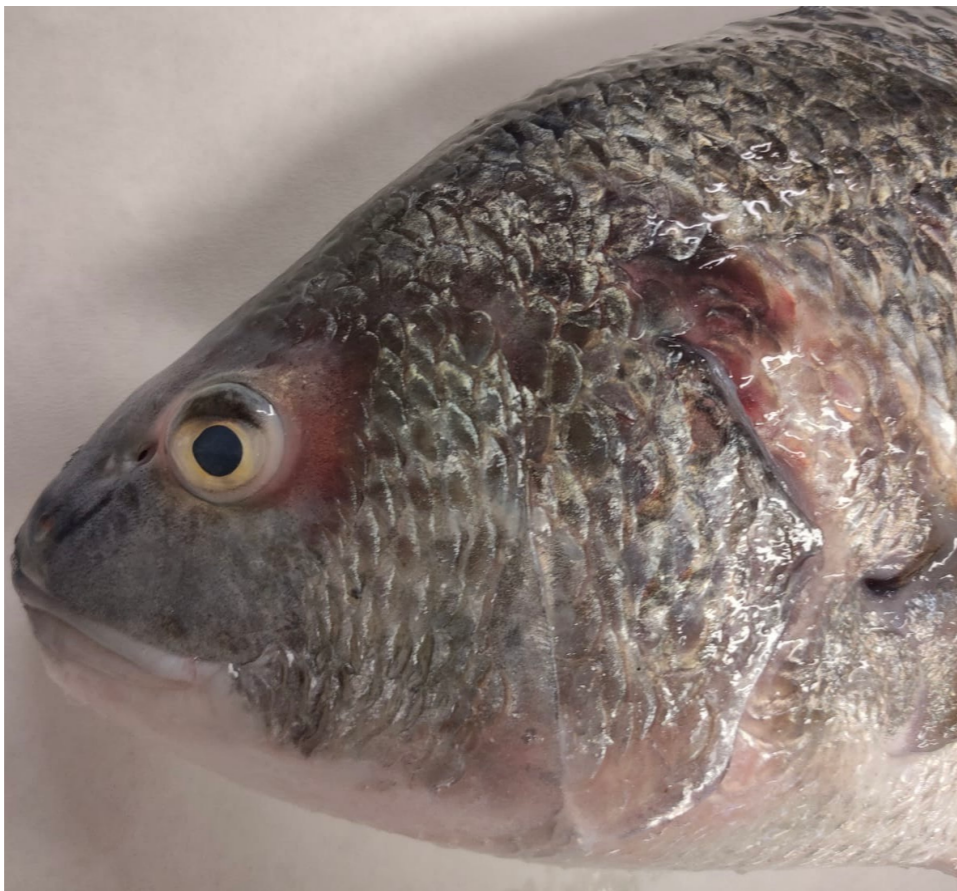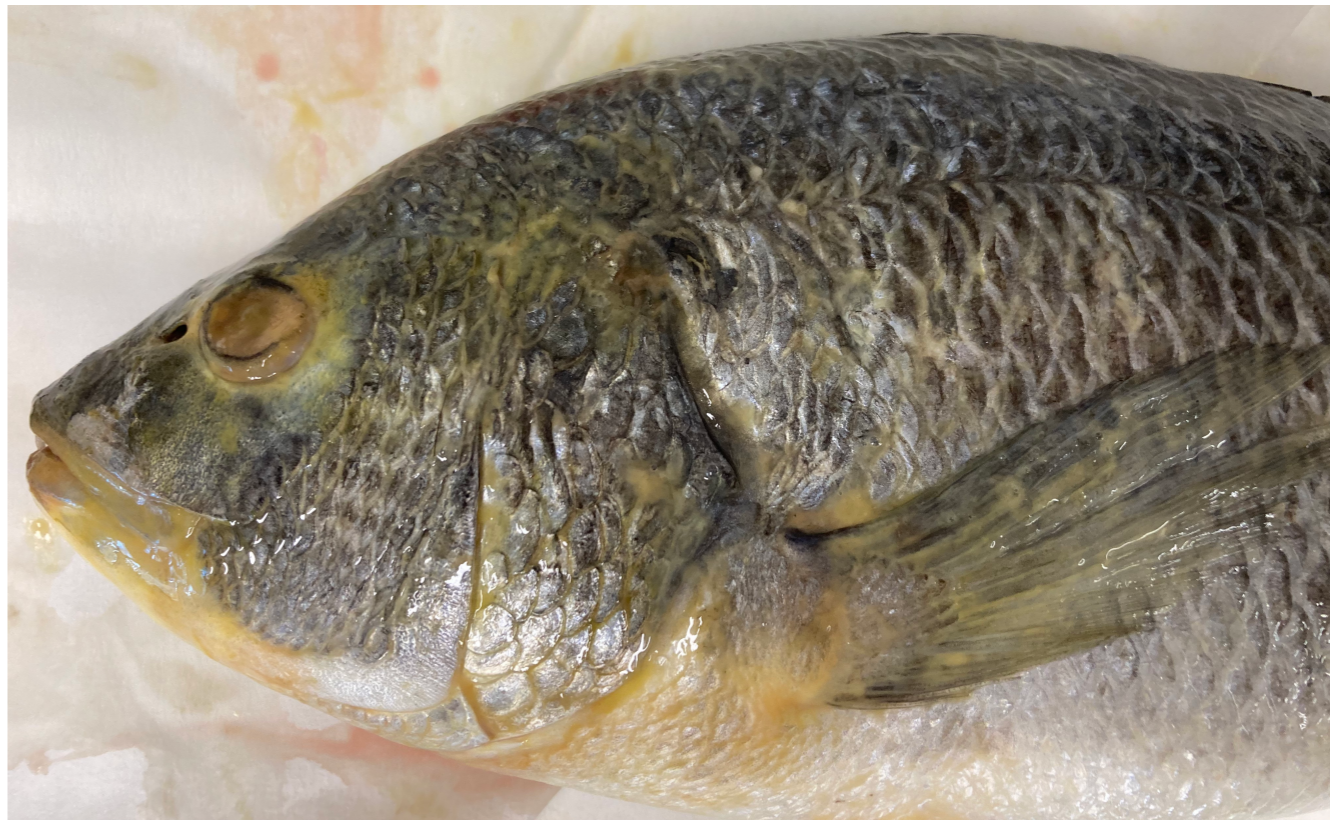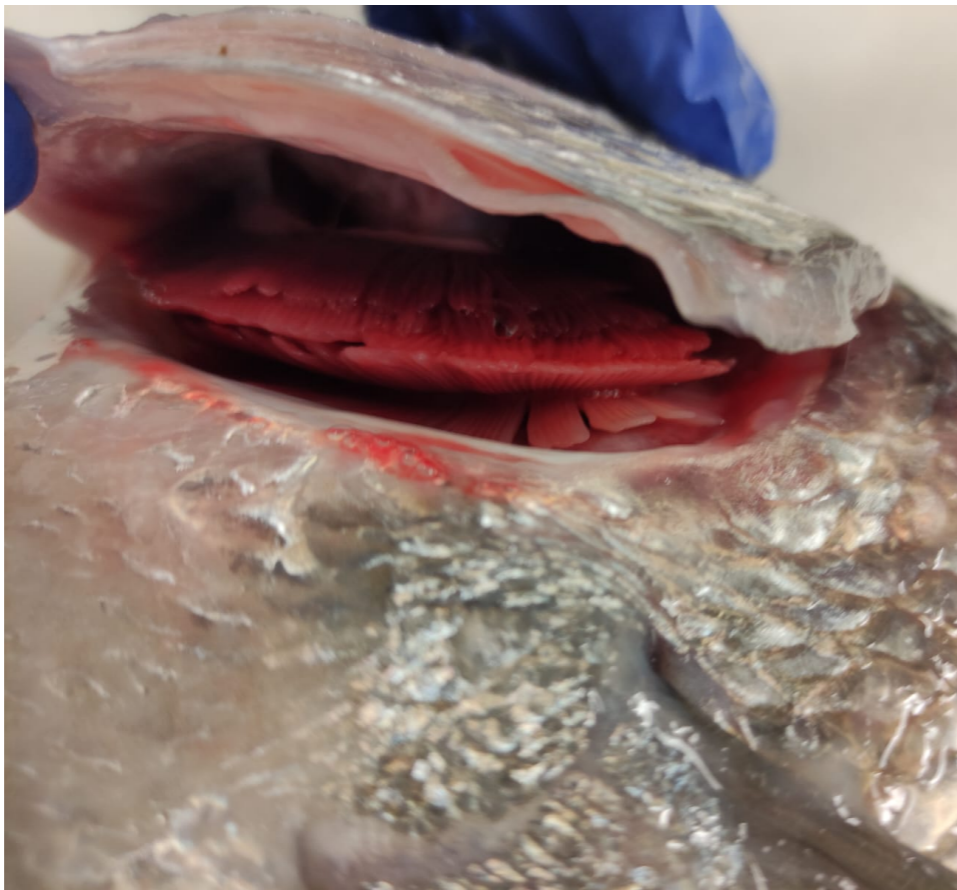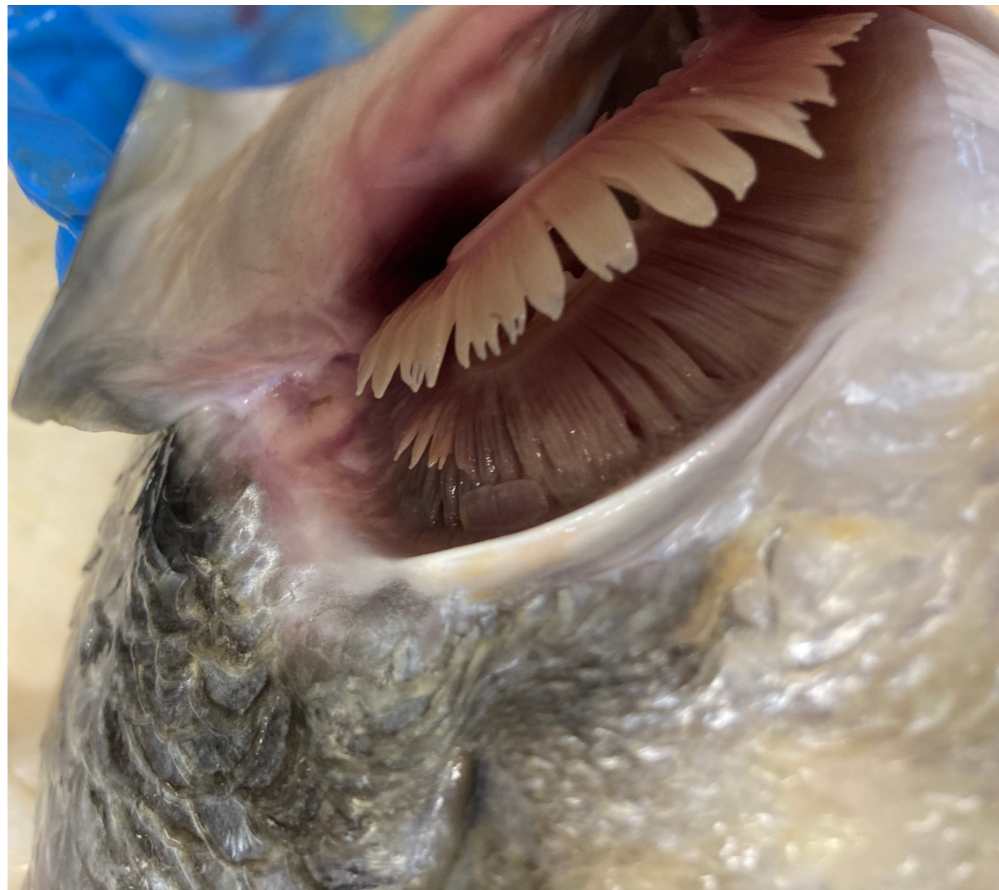

**sobaity bream**

Supplement: Supplementary file 1 [file foods-14-00690-s001.zip › Figure S8 sobaity bream.pdf]
